# Supplementary material for: Polyketide synthase gene clusters in the ericoid mycorrhizal fungus Oidiodendron maius: genome-wide prediction, expression profiling, and functional characterization of knockout mutants
Source: Front Plant Sci. 2026 Sep 16;17:1924430. doi: 10.3389/fpls.2026.1924430 (PMC13623903; doi:10.3389/fpls.2026.1924430)
Supplement: Supplementary file 1 [file SupplementaryFile1.zip › Supplementary Materials/Supplementary Folder 1.zip!/Oidma1_AssemblyScaffolds_Repeatmasked/knownclusterblast/cluster28/jgi.p_Oidma1_182120_mibig_hits.html]

| MIBiG Protein | Description | MIBiG Cluster | MiBiG Product | % ID | % Coverage | BLAST Score | E-value |
| --- | --- | --- | --- | --- | --- | --- | --- |
| BAV19379.1 | polyketide\_synthase | BGC0001390 | LL-Z1272beta\_biosynthetic\_gene\_cluster | 39 | 71.7848791894 | 1250 | 0.0 |
| AGN71604.1 | conidial\_yellow\_pigment\_biosynthesis\_polyketide\_synthase | BGC0000027 | Azaphilone\_biosynthetic\_gene\_cluster | 31 | 88.7763055339 | 1044 | 1.6e-304 |
| ADH01663.1 | putative\_polyketide\_synthase\_PKS3 | BGC0000099 | Monascorubrin\_biosynthetic\_gene\_cluster | 31 | 88.8932190179 | 1028 | 1.6e-299 |
| EED18001.1 | polyketide\_synthase,\_putative | BGC0000154 | Stipitatic\_acid\_biosynthetic\_gene\_cluster | 30 | 89.3998441154 | 955 | 1e-277 |
| CBF69451.1 | polyketide\_synthase,\_putative\_(JCVI) | BGC0000037 | Cichorine\_biosynthetic\_gene\_cluster | 32 | 72.3304754482 | 867 | 4.7e-251 |
| ALA62323.1 | methylphloroacetophenone\_synthase | BGC0001309 | Usnic\_acid\_biosynthetic\_gene\_cluster | 33 | 68.5113016368 | 860 | 4.4e-249 |
| CDM36726.1 | Beta-ketoacyl\_synthase | BGC0001360 | Mycophenolic\_acid\_biosynthetic\_gene\_cluster | 33 | 67.7708495713 | 839 | 1e-242 |
| CBF80428.1 | polyketide\_synthase,\_putative\_(JCVI) | BGC0000680 | Austinol/dehydroaustinol,\_locus\_1\_biosynthetic\_gene\_cluster | 32 | 69.9532346064 | 822 | 1.7e-237 |
| ADY00130.1 | polyketide\_synthase | BGC0000104 | Mycophenolic\_acid\_biosynthetic\_gene\_cluster | 33 | 58.6515978176 | 800 | 4.1e-231 |
| ALI92655.1 | CitS\_citrinin\_polyketide\_synthase | BGC0001338 | Citrinin\_biosynthetic\_gene\_cluster | 30 | 68.082618862 | 782 | 1.5e-225 |
| EHA28237.1 | hypothetical\_protein | BGC0001143 | Azanigerone\_biosynthetic\_gene\_cluster | 30 | 70.5378020265 | 772 | 1.2e-222 |
| CAP95404.1 |  | BGC0001404 | Sorbicillin\_biosynthetic\_gene\_cluster | 29 | 71.8628215121 | 693 | 9.3e-199 |
| EAA65602.1 | hypothetical\_protein | BGC0000022 | Asperfuranone\_biosynthetic\_gene\_cluster | 32 | 52.7669524552 | 664 | 6e-190 |
| EAQ86392.1 | hypothetical\_protein | BGC0001405 | Chaetoviridin\_/\_chaetomugilin\_biosynthetic\_gene\_cluster | 33 | 44.855806703 | 602 | 1.7e-171 |
| ACD39762.1 | non-reducing\_polyketide\_synthase | BGC0000077 | Hypothemycin\_biosynthetic\_gene\_cluster | 29 | 55.2221356196 | 503 | 1.8e-141 |
| ACD39753.1 | non-reducing\_polyketide\_synthase | BGC0000076 | Hypothemycin\_biosynthetic\_gene\_cluster | 28 | 55.2221356196 | 502 | 3e-141 |
| ABU63483.1 | polyketide\_synthase | BGC0001272 | Elsinochrome\_B\_/\_elsinochrome\_C\_biosynthetic\_gene\_cluster | 28 | 61.8472330475 | 500 | 1.5e-140 |
| ADM79459.1 | PKS16\_protein | BGC0001266 | Grayanic\_acid\_biosynthetic\_gene\_cluster | 26 | 68.3164458301 | 497 | 9.8e-140 |
| ABM21570.1 | CrpB | BGC0000975 | Cryptophycin\_biosynthetic\_gene\_cluster | 31 | 49.2985190959 | 495 | 3.7e-139 |
| CBF74114.1 | Conidial\_yellow\_pigment\_biosynthesis\_polyketide\_synthase\_(PKS)(EC\_2.3.1.-)\_[Source:UniProtKB/Swiss-Prot;Acc:Q03149] | BGC0000107 | Naphthopyrone\_biosynthetic\_gene\_cluster | 28 | 57.8721745908 | 494 | 6.3e-139 |
| BAD22832.1 | polyketide\_synthase | BGC0001265 | Melanin\_biosynthetic\_gene\_cluster | 27 | 71.4341387373 | 490 | 9.1e-138 |
| ABB90282.1 | polyketide\_synthase | BGC0001057 | Zearalenone\_biosynthetic\_gene\_cluster | 27 | 56.1184723305 | 483 | 1.1e-135 |
| ADI24926.1 | VrtA | BGC0000168 | Viridicatumtoxin\_biosynthetic\_gene\_cluster | 28 | 63.1722525331 | 480 | 1.2e-134 |
| CCE67070.1 | polyketide\_synthase | BGC0001242 | Fusarubin\_biosynthetic\_gene\_cluster | 27 | 68.0436477007 | 478 | 6.1e-134 |
| EAU29529.1 | hypothetical\_protein | BGC0000682 | Terretonin\_biosynthetic\_gene\_cluster | 36 | 32.3070927514 | 477 | 8e-134 |
| AIW82279.1 | PuwB | BGC0001125 | Puwainaphycins\_biosynthetic\_gene\_cluster | 32 | 41.4653156664 | 477 | 8e-134 |
| ACR33078.1 | polyketide\_synthase | BGC0000017 | Anatoxin\_biosynthetic\_gene\_cluster | 30 | 47.9734996103 | 475 | 3e-133 |
| CBD77748.1 | polyketide\_synthase | BGC0000974 | Crocacin\_biosynthetic\_gene\_cluster | 33 | 45.7911145752 | 475 | 3e-133 |
| CCT67991.1 | bikaverin\_cluster-polyketide\_synthase | BGC0000030 | Bikaverin\_biosynthetic\_gene\_cluster | 27 | 69.2907248636 | 474 | 8.9e-133 |
| EAU38791.1 | hypothetical\_protein | BGC0000161 | Terrein\_biosynthetic\_gene\_cluster | 28 | 58.1839438815 | 473 | 1.2e-132 |
| AAT70103.1 | CurH | BGC0001165 | Curacin\_biosynthetic\_gene\_cluster | 34 | 35.2299298519 | 471 | 7.5e-132 |
| EGD99348.1 | polyketide\_synthase | BGC0001144 | Neosartoricin\_biosynthetic\_gene\_cluster | 33 | 35.3858144973 | 471 | 7.5e-132 |
| AEE88282.1 | CurH | BGC0000976 | Curacin\_biosynthetic\_gene\_cluster | 34 | 35.2299298519 | 471 | 7.5e-132 |
| ABX60153.1 | polyketide\_synthase | BGC0000978 | Cylindrospermopsin\_biosynthetic\_gene\_cluster | 34 | 35.0350740452 | 467 | 1.1e-130 |
| AAN59953.1 | polyketide\_synthase\_1 | BGC0001258 | 1,8-dihydroxynaphthalene\_biosynthetic\_gene\_cluster | 27 | 64.302416212 | 465 | 4.1e-130 |
| ADF88275.1 | polyketide\_synthase | BGC0000981 | Cylindrospermopsin\_biosynthetic\_gene\_cluster | 35 | 35.2689010133 | 465 | 4.1e-130 |
| AHV78253.1 | ResS2 | BGC0001246 | Trans-resorcylide\_biosynthetic\_gene\_cluster | 27 | 61.4575214341 | 464 | 9.2e-130 |
| CAQ18829.1 | polyketide\_synthase | BGC0000954 | Ajudazol\_biosynthetic\_gene\_cluster | 36 | 35.0740452065 | 463 | 1.2e-129 |
| CAD19087.1 | StiC\_protein | BGC0000153 | Stigmatellin\_biosynthetic\_gene\_cluster | 33 | 38.3865939205 | 462 | 3.5e-129 |
| AEU11006.1 | NpnB | BGC0001029 | Nostophycin\_biosynthetic\_gene\_cluster | 34 | 37.3733437256 | 461 | 7.8e-129 |
| ADF88280.1 | polyketide\_synthase | BGC0000981 | Cylindrospermopsin\_biosynthetic\_gene\_cluster | 33 | 36.0483242401 | 460 | 1.7e-128 |
| CCH26295.1 | putative\_polyketide\_synthase | BGC0001158\_c6 | Bikaverin\_biosynthetic\_gene\_cluster | 28 | 64.1465315666 | 456 | 1.5e-127 |
| ABX60162.1 | polyketide\_synthase | BGC0000978 | Cylindrospermopsin\_biosynthetic\_gene\_cluster | 33 | 35.7755261107 | 456 | 1.5e-127 |
| AAK57187.1 | MxaC | BGC0001022 | Myxalamid\_biosynthetic\_gene\_cluster | 33 | 36.7498051442 | 456 | 1.9e-127 |
| AIW82282.1 | PuwE | BGC0001125 | Puwainaphycins\_biosynthetic\_gene\_cluster | 35 | 35.2689010133 | 456 | 2.5e-127 |
| AEU11005.1 | NpnA | BGC0001029 | Nostophycin\_biosynthetic\_gene\_cluster | 32 | 39.8674980514 | 454 | 9.5e-127 |
| AAS98782.1 | JamK | BGC0001001 | Jamaicamide\_biosynthetic\_gene\_cluster | 35 | 33.0475448168 | 453 | 2.1e-126 |
| AMB48442.1 | polyketide\_synthase | BGC0001357 | Carbamidocyclophane\_biosynthetic\_gene\_cluster | 33 | 35.3078721746 | 452 | 2.8e-126 |
| ACD39770.1 | non-reducing\_polyketide\_synthase | BGC0000134 | Radicicol\_biosynthetic\_gene\_cluster | 28 | 53.1176929072 | 451 | 4.7e-126 |
| AFV96138.1 | polyketide\_synthase | BGC0001064 | Cylindrocyclophane\_biosynthetic\_gene\_cluster | 33 | 35.3078721746 | 451 | 4.7e-126 |
| AAT70107.1 | CurL | BGC0001165 | Curacin\_biosynthetic\_gene\_cluster | 33 | 35.8534684334 | 450 | 1.4e-125 |
| AEE88278.1 | CurL | BGC0000976 | Curacin\_biosynthetic\_gene\_cluster | 33 | 35.8534684334 | 450 | 1.4e-125 |
| AEN83889.1 | AdaA | BGC0000156 | TAN-1612\_biosynthetic\_gene\_cluster | 29 | 50.8963367108 | 448 | 4e-125 |
| AAO62584.1 | polyketide\_synthase\_type\_1 | BGC0001016 | Microcystin\_biosynthetic\_gene\_cluster | 31 | 44.3102104443 | 448 | 4e-125 |
| CBF70387.1 | polyketide\_synthase,\_putative\_(JCVI) | BGC0000684 | Asperthecin\_biosynthetic\_gene\_cluster | 27 | 62.8604832424 | 448 | 5.2e-125 |
| AEE88289.1 | CurA | BGC0000976 | Curacin\_biosynthetic\_gene\_cluster | 32 | 36.8667186282 | 448 | 6.8e-125 |
| AAT70096.1 | CurA | BGC0001165 | Curacin\_biosynthetic\_gene\_cluster | 32 | 36.8667186282 | 448 | 6.8e-125 |
| AEE88281.1 | CurI | BGC0000976 | Curacin\_biosynthetic\_gene\_cluster | 33 | 36.0483242401 | 446 | 2e-124 |
| AAT70104.1 | CurI | BGC0001165 | Curacin\_biosynthetic\_gene\_cluster | 33 | 36.0483242401 | 446 | 2e-124 |
| AHV78247.1 | LasS2 | BGC0001245 | Lasiodiplodin\_biosynthetic\_gene\_cluster | 28 | 53.7022603274 | 446 | 2e-124 |
| AAF19814.1 | MtaF | BGC0001024 | Myxothiazol\_biosynthetic\_gene\_cluster | 34 | 36.5159781761 | 446 | 2.6e-124 |
| AAS98777.1 | JamE | BGC0001001 | Jamaicamide\_biosynthetic\_gene\_cluster | 33 | 35.4247856586 | 445 | 4.4e-124 |
| AHB82053.1 | polyketide\_synthase | BGC0001019 | Microsclerodermins\_biosynthetic\_gene\_cluster | 34 | 35.1519875292 | 445 | 4.4e-124 |
| AHB82064.1 | polyketide\_synthase | BGC0001231 | Microsclerodermins\_biosynthetic\_gene\_cluster | 34 | 35.0350740452 | 443 | 1.3e-123 |
| AGC95321.1 | CurS2 | BGC0000045 | Dehydrocurvularin\_biosynthetic\_gene\_cluster | 27 | 55.2221356196 | 443 | 2.2e-123 |
| ADN13832.1 | Beta-ketoacyl\_synthase | BGC0001164 | 1-heptadecene\_biosynthetic\_gene\_cluster | 33 | 34.9571317225 | 442 | 2.9e-123 |
| AGC45622.1 | polyketide\_synthase | BGC0001394 | Phenalamide\_biosynthetic\_gene\_cluster | 30 | 49.6492595479 | 442 | 3.7e-123 |
| AKN45693.1 | polyketide\_synthase | BGC0001284 | Alternariol\_biosynthetic\_gene\_cluster | 31 | 45.7131722525 | 441 | 4.9e-123 |
| CAO98879.1 | polyketide\_synthase\_AufD | BGC0000023 | Aurafuron\_biosynthetic\_gene\_cluster | 32 | 39.6336710834 | 441 | 6.4e-123 |
| ACR50791.1 | putative\_polyketide\_synthase | BGC0000163 | Tetronasin\_biosynthetic\_gene\_cluster | 32 | 37.763055339 | 440 | 1.1e-122 |
| AAS98783.1 | JamL | BGC0001001 | Jamaicamide\_biosynthetic\_gene\_cluster | 33 | 35.697583788 | 439 | 2.4e-122 |
| AHA12079.1 | polyketide\_synthase\_type\_1 | BGC0001172 | Chlorizidine\_A\_biosynthetic\_gene\_cluster | 33 | 35.4247856586 | 439 | 3.2e-122 |
| ADI24953.1 | GsfA | BGC0000070 | Griseofulvin\_biosynthetic\_gene\_cluster | 31 | 41.8160561185 | 438 | 4.1e-122 |
| EAL89339.1 | polyketide\_synthase,\_putative | BGC0001403 | Trypacidin\_biosynthetic\_gene\_cluster | 27 | 58.2618862042 | 437 | 1.2e-121 |
| CAD19090.1 | StiF\_protein | BGC0000153 | Stigmatellin\_biosynthetic\_gene\_cluster | 30 | 48.0124707716 | 436 | 1.6e-121 |
| BAD38874.1 | polyketide\_synthase | BGC0000111 | Neocarzilin\_biosynthetic\_gene\_cluster | 34 | 35.3078721746 | 436 | 1.6e-121 |
| AAD38786.1 | polyketide\_synthase | BGC0001257 | 1,3,6,8-tetrahydroxynaphthalene\_biosynthetic\_gene\_cluster | 26 | 72.6422447389 | 436 | 2e-121 |
| ADF88276.1 | polyketide\_synthase | BGC0000981 | Cylindrospermopsin\_biosynthetic\_gene\_cluster | 32 | 35.580670304 | 436 | 2.7e-121 |
| CAD19091.1 | StiG\_protein | BGC0000153 | Stigmatellin\_biosynthetic\_gene\_cluster | 29 | 53.429462198 | 435 | 6e-121 |
| EAA59563.1 | hypothetical\_protein | BGC0000057 | F9775\_biosynthetic\_gene\_cluster | 26 | 68.1605611847 | 433 | 1.3e-120 |
| AAF00959.1 | McyD | BGC0001017 | Microcystin\_biosynthetic\_gene\_cluster | 34 | 34.7622759158 | 432 | 3e-120 |
| ABX60152.1 | polyketide\_synthase | BGC0000978 | Cylindrospermopsin\_biosynthetic\_gene\_cluster | 32 | 35.580670304 | 432 | 3.9e-120 |
| CAD89777.1 | MelF\_protein | BGC0001009 | Melithiazol\_biosynthetic\_gene\_cluster | 33 | 36.8277474669 | 432 | 3.9e-120 |
| ACR33077.1 | polyketide\_synthase | BGC0000017 | Anatoxin\_biosynthetic\_gene\_cluster | 30 | 40.2182385035 | 431 | 6.6e-120 |
| AID65222.1 | putative\_aspartate\_racemase | BGC0000335 | Cystomanamides\_biosynthetic\_gene\_cluster | 34 | 34.9571317225 | 431 | 6.6e-120 |
| CAD29793.1 | polyketide\_synthase\_type\_I | BGC0001015 | Microcystin\_biosynthetic\_gene\_cluster | 32 | 40.0623538581 | 429 | 2.5e-119 |
| CCE88376.1 | polyketide\_synthase | BGC0001034 | Pellasoren\_biosynthetic\_gene\_cluster | 30 | 48.0124707716 | 428 | 4.3e-119 |
| AGC45619.1 | polyketide\_synthase | BGC0001394 | Phenalamide\_biosynthetic\_gene\_cluster | 31 | 45.2844894778 | 428 | 5.6e-119 |
| AAW03329.1 | CtaF | BGC0000982 | Cystothiazole\_A\_biosynthetic\_gene\_cluster | 34 | 36.9446609509 | 428 | 5.6e-119 |
| CAD19092.1 | StiH\_protein | BGC0000153 | Stigmatellin\_biosynthetic\_gene\_cluster | 32 | 35.0350740452 | 427 | 9.5e-119 |
| CAD19093.1 | StiJ\_protein | BGC0000153 | Stigmatellin\_biosynthetic\_gene\_cluster | 33 | 35.7755261107 | 426 | 1.6e-118 |
| EAL84397.1 | polyketide\_synthase,\_putative | BGC0001118 | Endocrocin\_biosynthetic\_gene\_cluster | 33 | 35.6586126267 | 426 | 2.1e-118 |
| AGC45620.1 | polyketide\_synthase | BGC0001394 | Phenalamide\_biosynthetic\_gene\_cluster | 32 | 34.9571317225 | 425 | 4.7e-118 |
| ABX60163.1 | polyketide\_synthase | BGC0000978 | Cylindrospermopsin\_biosynthetic\_gene\_cluster | 32 | 38.5814497272 | 425 | 4.7e-118 |
| CAD19086.1 | StiB\_protein | BGC0000153 | Stigmatellin\_biosynthetic\_gene\_cluster | 32 | 35.0740452065 | 425 | 4.7e-118 |
| ADF88277.1 | polyketide\_synthase | BGC0000981 | Cylindrospermopsin\_biosynthetic\_gene\_cluster | 32 | 38.5814497272 | 425 | 6.2e-118 |
| AHH34186.1 | polyketide\_synthase | BGC0001161 | 1-heptadecene\_biosynthetic\_gene\_cluster | 32 | 36.2042088854 | 424 | 8.1e-118 |
| CBF90097.1 | hypothetical\_protein\_similar\_to\_polyketide\_synthase\_(Broad) | BGC0001271 | Emericellin\_biosynthetic\_gene\_cluster | 33 | 35.2689010133 | 424 | 1.1e-117 |
| AAY42396.1 | HctD | BGC0001000 | Hectochlorin\_biosynthetic\_gene\_cluster | 33 | 35.3858144973 | 423 | 1.8e-117 |
| ACA99172.1 | Polyketide\_synthase | BGC0001160 | Nonadecene\_biosynthetic\_gene\_cluster | 33 | 35.0740452065 | 422 | 4e-117 |
| EED53479.1 | polyketide\_synthase,\_putative | BGC0001304 | Aflavarin\_biosynthetic\_gene\_cluster | 30 | 49.766173032 | 421 | 5.2e-117 |
| CAQ18835.1 | polyketide\_synthase | BGC0000954 | Ajudazol\_biosynthetic\_gene\_cluster | 32 | 38.4255650818 | 421 | 5.2e-117 |
| AGO59040.1 | PtaA | BGC0000121 | Pestheic\_acid\_biosynthetic\_gene\_cluster | 29 | 47.4279033515 | 421 | 6.8e-117 |
| AEP40936.1 | polyketide\_synthase\_type\_I | BGC0000021 | Apoptolidin\_biosynthetic\_gene\_cluster | 31 | 43.6866718628 | 421 | 8.9e-117 |
| AEE88279.1 | CurK | BGC0000976 | Curacin\_biosynthetic\_gene\_cluster | 28 | 51.597817615 | 420 | 2e-116 |
| AAT70106.1 | CurK | BGC0001165 | Curacin\_biosynthetic\_gene\_cluster | 28 | 51.597817615 | 420 | 2e-116 |
| ACR33079.1 | polyketide\_synthase | BGC0000017 | Anatoxin\_biosynthetic\_gene\_cluster | 33 | 35.3468433359 | 418 | 7.5e-116 |
| AEU17899.1 | putative\_type\_I\_PKS | BGC0001072 | Simocyclinone\_biosynthetic\_gene\_cluster | 32 | 37.295401403 | 417 | 1.3e-115 |
| CBD77738.1 | polyketide\_synthase | BGC0000974 | Crocacin\_biosynthetic\_gene\_cluster | 33 | 35.5027279813 | 416 | 1.7e-115 |
| AFU82615.1 | polyketide\_synthase | BGC0000998 | Guadinomine\_biosynthetic\_gene\_cluster | 33 | 34.6843335931 | 416 | 1.7e-115 |
| AAF26921.1 | polyketide\_synthase | BGC0000988 | Epothilone\_biosynthetic\_gene\_cluster | 34 | 34.9961028839 | 416 | 2.9e-115 |
| AAF62883.1 | epoD | BGC0000991 | Epothilone\_biosynthetic\_gene\_cluster | 30 | 41.0366328917 | 416 | 2.9e-115 |
| CAQ18828.1 | polyketide\_synthase | BGC0000954 | Ajudazol\_biosynthetic\_gene\_cluster | 34 | 35.8924395947 | 415 | 3.7e-115 |
| ACB46195.1 | polyketide\_synthase | BGC0000989 | Epothilone\_biosynthetic\_gene\_cluster | 34 | 34.9961028839 | 414 | 8.3e-115 |
| CAQ34928.1 | polyketide\_synthase | BGC0000986 | Dkxanthene\_biosynthetic\_gene\_cluster | 29 | 48.4411535464 | 414 | 8.3e-115 |
| ADB12491.1 | EpoD | BGC0000990 | Epothilone\_biosynthetic\_gene\_cluster | 34 | 34.9961028839 | 414 | 8.3e-115 |
| AEP40939.1 | polyketide\_synthase\_type\_I | BGC0000021 | Apoptolidin\_biosynthetic\_gene\_cluster | 32 | 38.4255650818 | 413 | 2.4e-114 |
| AGC24270.1 | PrlP | BGC0001038 | Pyralomicin\_biosynthetic\_gene\_cluster | 32 | 35.6196414653 | 412 | 3.2e-114 |
| AEZ64504.1 | Herc | BGC0001065 | Herboxidiene\_biosynthetic\_gene\_cluster | 32 | 40.7248636009 | 412 | 3.2e-114 |
| CAJ88177.1 | putative\_type\_I\_polyketide\_synthase | BGC0000151 | Stambomycin\_biosynthetic\_gene\_cluster | 33 | 36.0093530787 | 412 | 4.1e-114 |
| ADH04660.1 | TugD | BGC0001342 | Thuggacin\_biosynthetic\_gene\_cluster | 33 | 36.0093530787 | 411 | 7.1e-114 |
| ABO15860.1 | polyketide\_synthase | BGC0000130 | Pyrrolomycin\_biosynthetic\_gene\_cluster | 34 | 32.5409197194 | 411 | 9.2e-114 |
| ABC84471.1 | NigAVII | BGC0000114 | Nigericin\_biosynthetic\_gene\_cluster | 32 | 35.4247856586 | 411 | 9.2e-114 |
| AAK19883.1 | soraphen\_polyketide\_synthase\_A | BGC0000147 | Soraphen\_biosynthetic\_gene\_cluster | 31 | 40.1402961808 | 411 | 9.2e-114 |
| AHH99925.1 | PKS\_I | BGC0000002 | Aculeximycin\_biosynthetic\_gene\_cluster | 32 | 37.6071706937 | 410 | 1.2e-113 |
| AGC45624.1 | polyketide\_synthase | BGC0001394 | Phenalamide\_biosynthetic\_gene\_cluster | 29 | 48.051441933 | 410 | 1.6e-113 |
| AEE88284.1 | CurF | BGC0000976 | Curacin\_biosynthetic\_gene\_cluster | 27 | 53.6243180047 | 409 | 3.5e-113 |
| AHA38201.1 | GphH | BGC0000069 | Gephyronic\_acid\_biosynthetic\_gene\_cluster | 33 | 35.580670304 | 409 | 3.5e-113 |
| AAC46025.1 | polyketide\_synthase\_module\_3 | BGC0000113 | Niddamycin\_biosynthetic\_gene\_cluster | 30 | 43.8035853468 | 409 | 3.5e-113 |
| CAQ43076.1 | polyketide\_synthase | BGC0000970 | Chondrochloren\_biosynthetic\_gene\_cluster | 29 | 44.3491816056 | 408 | 4.6e-113 |
| BAJ16467.1 | polyketide\_synthase | BGC0000058 | FD-891\_biosynthetic\_gene\_cluster | 30 | 44.1543257989 | 408 | 6e-113 |
| AAT70101.1 | CurF | BGC0001165 | Curacin\_biosynthetic\_gene\_cluster | 27 | 53.6243180047 | 408 | 7.8e-113 |
| ADM46357.1 | polyketide\_synthase | BGC0000106 | Naphthomycin\_biosynthetic\_gene\_cluster | 33 | 35.7365549493 | 408 | 7.8e-113 |
| CAQ43078.1 | polyketide\_synthase | BGC0000970 | Chondrochloren\_biosynthetic\_gene\_cluster | 32 | 39.2049883087 | 407 | 1e-112 |
| AIT55259.1 | GulB | BGC0000072 | Gulmirecin\_biosynthetic\_gene\_cluster | 33 | 35.3078721746 | 407 | 1e-112 |
| BAT51065.1 | type\_I\_polyketide\_synthase | BGC0001296 | Streptazone\_E\_biosynthetic\_gene\_cluster | 31 | 39.6726422447 | 407 | 1.3e-112 |
| AAU04878.1 | polyketide\_synthase | BGC0000365 | Glycopeptidolipid\_biosynthetic\_gene\_cluster | 31 | 36.0093530787 | 407 | 1.3e-112 |
| CAQ64692.1 | lasalocid\_modular\_polyketide\_synthase | BGC0000087 | Lasalocid\_biosynthetic\_gene\_cluster | 33 | 34.8402182385 | 407 | 1.3e-112 |
| CAL58687.1 | polyketide\_synthase | BGC0000149 | Spirangien\_biosynthetic\_gene\_cluster | 30 | 45.1286048324 | 406 | 1.7e-112 |
| AEE88277.1 | CurM | BGC0000976 | Curacin\_biosynthetic\_gene\_cluster | 32 | 35.3078721746 | 406 | 1.7e-112 |
| ACV42478.1 | polyketide\_synthase | BGC0000043 | Curacin\_A\_biosynthetic\_gene\_cluster | 32 | 35.3078721746 | 406 | 1.7e-112 |
| AAT70108.1 | CurM | BGC0001165 | Curacin\_biosynthetic\_gene\_cluster | 32 | 35.3078721746 | 406 | 1.7e-112 |
| AAW03328.1 | CtaE | BGC0000982 | Cystothiazole\_A\_biosynthetic\_gene\_cluster | 33 | 35.3468433359 | 406 | 1.7e-112 |
| CCE88380.1 | polyketide\_synthase | BGC0001034 | Pellasoren\_biosynthetic\_gene\_cluster | 29 | 48.0904130943 | 406 | 2.3e-112 |
| AAC49191.1 | putative\_polyketide\_synthase | BGC0000152 | Sterigmatocystin\_biosynthetic\_gene\_cluster | 29 | 43.1410756041 | 406 | 3e-112 |
| AAC38076.1 | polyketide\_synthase\_type\_I | BGC0000127 | Pyoluteorin\_biosynthetic\_gene\_cluster | 32 | 35.3858144973 | 406 | 3e-112 |
| AAK57188.1 | MxaD | BGC0001022 | Myxalamid\_biosynthetic\_gene\_cluster | 28 | 52.3382696804 | 405 | 3.9e-112 |
| AAF26922.1 | polyketide\_synthase | BGC0000988 | Epothilone\_biosynthetic\_gene\_cluster | 29 | 45.2455183164 | 405 | 3.9e-112 |
| AAF62884.1 | EpoE | BGC0000991 | Epothilone\_biosynthetic\_gene\_cluster | 29 | 45.2455183164 | 405 | 3.9e-112 |
| CCE88381.1 | polyketide\_synthase | BGC0001034 | Pellasoren\_biosynthetic\_gene\_cluster | 29 | 48.285268901 | 405 | 5.1e-112 |
| ADB12492.1 | EpoE | BGC0000990 | Epothilone\_biosynthetic\_gene\_cluster | 29 | 45.2455183164 | 405 | 5.1e-112 |
| AHH99920.1 | PKS\_I | BGC0000002 | Aculeximycin\_biosynthetic\_gene\_cluster | 32 | 34.3335931411 | 405 | 5.1e-112 |
| CAL58686.1 | polyketide\_synthase | BGC0000149 | Spirangien\_biosynthetic\_gene\_cluster | 32 | 36.5549493375 | 404 | 6.6e-112 |
| BAC68129.1 | modular\_polyketide\_synthase | BGC0000059 | Filipin\_biosynthetic\_gene\_cluster | 31 | 39.8285268901 | 404 | 6.6e-112 |
| ABB88521.1 | polyketide\_synthase\_type\_I | BGC0000050 | E-837\_biosynthetic\_gene\_cluster | 31 | 39.2829306313 | 404 | 6.6e-112 |
| AIT55260.1 | GulC | BGC0000072 | Gulmirecin\_biosynthetic\_gene\_cluster | 31 | 40.1402961808 | 404 | 6.6e-112 |
| ACH72912.1 | AflC | BGC0000011 | Aflatoxin/sterigmatocystin\_biosynthetic\_gene\_cluster | 29 | 46.1028838659 | 404 | 6.6e-112 |
| ADZ24996.1 | polyketide\_synthase | BGC0000380 | Leupyrrin\_biosynthetic\_gene\_cluster | 30 | 48.7139516758 | 404 | 8.6e-112 |
| CAO98847.1 | polyketide\_synthase\_AufC | BGC0000023 | Aurafuron\_biosynthetic\_gene\_cluster | 32 | 36.1262665627 | 404 | 8.6e-112 |
| BAT51066.1 | type\_I\_polyketide\_synthase | BGC0001296 | Streptazone\_E\_biosynthetic\_gene\_cluster | 33 | 35.6586126267 | 404 | 1.1e-111 |
| ADZ24997.1 | polyketide\_synthase | BGC0000380 | Leupyrrin\_biosynthetic\_gene\_cluster | 28 | 48.9867498051 | 403 | 1.5e-111 |
| AIT55261.1 | GulD | BGC0000072 | Gulmirecin\_biosynthetic\_gene\_cluster | 32 | 35.2689010133 | 403 | 1.9e-111 |
| BAG85030.1 | putative\_polyketide\_synthase | BGC0000086 | Lasalocid\_biosynthetic\_gene\_cluster | 32 | 38.8932190179 | 403 | 2.5e-111 |
| CAQ64690.1 | lasalocid\_modular\_polyketide\_synthase | BGC0000087 | Lasalocid\_biosynthetic\_gene\_cluster | 32 | 38.8932190179 | 403 | 2.5e-111 |
| AAF19813.1 | MtaE | BGC0001024 | Myxothiazol\_biosynthetic\_gene\_cluster | 32 | 35.46375682 | 403 | 2.5e-111 |
| CAC22145.1 | putative\_type\_I\_polyketide\_synthase | BGC0000038 | Coelimycin\_biosynthetic\_gene\_cluster | 33 | 35.9703819174 | 402 | 3.3e-111 |
| ADH04639.1 | TgaA | BGC0001051 | Thuggacin\_biosynthetic\_gene\_cluster | 33 | 35.697583788 | 402 | 4.3e-111 |
| ABK32258.1 | AmbD | BGC0000014 | Ambruticin\_biosynthetic\_gene\_cluster | 31 | 38.9321901793 | 402 | 4.3e-111 |
| BAB69192.1 | modular\_polyketide\_synthase | BGC0000117 | Oligomycin\_biosynthetic\_gene\_cluster | 31 | 44.6999220577 | 402 | 4.3e-111 |
| BAQ25483.1 | type\_I\_polyketide\_synthase | BGC0001288 | Maklamicin\_biosynthetic\_gene\_cluster | 32 | 35.3078721746 | 402 | 4.3e-111 |
| CAO98849.1 | polyketide\_synthase\_AufF | BGC0000023 | Aurafuron\_biosynthetic\_gene\_cluster | 31 | 35.3858144973 | 402 | 4.3e-111 |
| CAD19089.1 | StiE\_protein | BGC0000153 | Stigmatellin\_biosynthetic\_gene\_cluster | 31 | 34.8012470772 | 401 | 5.6e-111 |
| AAZ95017.1 | polyketide\_synthase | BGC0000048 | Dothistromin\_biosynthetic\_gene\_cluster | 32 | 35.0350740452 | 401 | 5.6e-111 |
| EAQ84779.1 | hypothetical\_protein | BGC0001219 | Shanorellin\_biosynthetic\_gene\_cluster | 34 | 34.4115354638 | 401 | 5.6e-111 |
| ADZ24998.1 | polyketide\_synthase | BGC0000380 | Leupyrrin\_biosynthetic\_gene\_cluster | 32 | 35.46375682 | 401 | 7.3e-111 |
| ABB88534.1 | polyketide\_synthase\_type\_I | BGC0000049 | E-492\_/\_E-975\_biosynthetic\_gene\_cluster | 32 | 39.1660171473 | 401 | 7.3e-111 |
| BAF02925.1 | type\_I\_polyketide\_synthase | BGC0000073 | Halstoctacosanolide\_biosynthetic\_gene\_cluster | 32 | 35.4247856586 | 401 | 7.3e-111 |
| ACO94483.1 | polyketide\_synthase\_type\_I | BGC0000097 | ML-449\_biosynthetic\_gene\_cluster | 31 | 37.9189399844 | 401 | 9.5e-111 |
| CAD19088.1 | StiD\_protein | BGC0000153 | Stigmatellin\_biosynthetic\_gene\_cluster | 34 | 36.0093530787 | 401 | 9.5e-111 |
| CAQ18833.1 | polyketide\_synthase | BGC0000954 | Ajudazol\_biosynthetic\_gene\_cluster | 33 | 35.580670304 | 401 | 9.5e-111 |
| AAF19812.1 | MtaD | BGC0001024 | Myxothiazol\_biosynthetic\_gene\_cluster | 29 | 49.5713172253 | 401 | 9.5e-111 |
| AAT69682.1 | polyketide\_synthase | BGC0001247 | Cercosporin\_biosynthetic\_gene\_cluster | 25 | 71.5510522214 | 401 | 9.5e-111 |
| AAK57186.1 | MxaB2 | BGC0001022 | Myxalamid\_biosynthetic\_gene\_cluster | 34 | 32.6968043648 | 400 | 1.6e-110 |
| AAX98188.1 | polyketide\_synthase\_type\_I | BGC0000052 | ECO-02301\_biosynthetic\_gene\_cluster | 31 | 39.0880748246 | 400 | 1.6e-110 |
| ACB46196.1 | polyketide\_synthase | BGC0000989 | Epothilone\_biosynthetic\_gene\_cluster | 29 | 45.4014029618 | 399 | 2.1e-110 |
| CAQ34919.1 | polyketide\_synthase | BGC0000986 | Dkxanthene\_biosynthetic\_gene\_cluster | 31 | 38.9711613406 | 399 | 2.1e-110 |
| ACO94456.1 | polyketide\_synthase\_type\_I | BGC0000029 | BE-14106\_biosynthetic\_gene\_cluster | 31 | 38.0748246298 | 399 | 2.1e-110 |
| ABI94379.1 | tautomycetin\_biosynthetic\_PKS | BGC0000157 | Tautomycetin\_biosynthetic\_gene\_cluster | 32 | 36.3990646921 | 399 | 2.8e-110 |
| AEP40940.1 | polyketide\_synthase\_type\_I | BGC0000021 | Apoptolidin\_biosynthetic\_gene\_cluster | 32 | 38.7373343726 | 399 | 2.8e-110 |
| BAQ21946.1 | putative\_type\_I\_polyketide\_synthase | BGC0001204 | Versipelostatin\_biosynthetic\_gene\_cluster | 31 | 40.0233826968 | 399 | 2.8e-110 |
| AAF00958.1 | McyE | BGC0001017 | Microcystin\_biosynthetic\_gene\_cluster | 32 | 35.46375682 | 399 | 2.8e-110 |
| AFU82616.1 | polyketide\_synthase | BGC0000998 | Guadinomine\_biosynthetic\_gene\_cluster | 31 | 36.1262665627 | 399 | 3.6e-110 |
| AHH99921.1 | PKS\_I | BGC0000002 | Aculeximycin\_biosynthetic\_gene\_cluster | 31 | 43.8425565082 | 399 | 3.6e-110 |
| AAX98191.1 | polyketide\_synthase\_type\_I | BGC0000052 | ECO-02301\_biosynthetic\_gene\_cluster | 31 | 41.6991426345 | 399 | 3.6e-110 |
| BAQ21939.1 | putative\_type\_I\_polyketide\_synthase | BGC0001204 | Versipelostatin\_biosynthetic\_gene\_cluster | 33 | 35.7365549493 | 399 | 3.6e-110 |
| AAZ77694.1 | ChlA2 | BGC0000036 | Chlorothricin\_biosynthetic\_gene\_cluster | 31 | 39.5167575994 | 398 | 4.7e-110 |
| ADM46360.1 | polyketide\_synthase | BGC0000106 | Naphthomycin\_biosynthetic\_gene\_cluster | 33 | 35.8534684334 | 398 | 4.7e-110 |
| BAJ16469.1 | polyketide\_synthase | BGC0000058 | FD-891\_biosynthetic\_gene\_cluster | 32 | 35.0350740452 | 398 | 6.2e-110 |
| ctg1\_orf523 |  | BGC0001199 | Akaeolide\_biosynthetic\_gene\_cluster | 32 | 36.3990646921 | 398 | 6.2e-110 |
| AAZ77693.1 | ChlA1 | BGC0000036 | Chlorothricin\_biosynthetic\_gene\_cluster | 33 | 35.7755261107 | 398 | 6.2e-110 |
| ABK32290.1 | JerD | BGC0000080 | Jerangolid\_biosynthetic\_gene\_cluster | 31 | 38.6593920499 | 398 | 6.2e-110 |
| AFL48520.1 | laidlomycin\_polyketide\_synthase\_(module\_9) | BGC0000084 | Laidlomycin\_biosynthetic\_gene\_cluster | 33 | 35.1519875292 | 398 | 6.2e-110 |
| AFV96142.1 | polyketide\_synthase | BGC0001064 | Cylindrocyclophane\_biosynthetic\_gene\_cluster | 31 | 35.1909586906 | 398 | 8.1e-110 |
| AAZ77699.1 | ChlA6 | BGC0000036 | Chlorothricin\_biosynthetic\_gene\_cluster | 32 | 35.5416991426 | 398 | 8.1e-110 |
| AMB48441.1 | polyketide\_synthase | BGC0001357 | Carbamidocyclophane\_biosynthetic\_gene\_cluster | 30 | 36.2042088854 | 398 | 8.1e-110 |
| AGY30676.1 | Ann4 | BGC0001298 | 4-Z-annimycin\_biosynthetic\_gene\_cluster | 31 | 40.6469212783 | 398 | 8.1e-110 |
| CAQ18839.1 | hybrid\_polyketide\_synthase/nonribosomal\_polypetide\_synthetase | BGC0000954 | Ajudazol\_biosynthetic\_gene\_cluster | 33 | 35.8924395947 | 398 | 8.1e-110 |
| BAG85026.1 | putative\_polyketide\_synthase | BGC0000086 | Lasalocid\_biosynthetic\_gene\_cluster | 32 | 39.8285268901 | 398 | 8.1e-110 |
| CAQ64686.1 | lasalocid\_modular\_polyketide\_synthase | BGC0000087 | Lasalocid\_biosynthetic\_gene\_cluster | 32 | 39.8285268901 | 398 | 8.1e-110 |
| AAC01710.1 | RifA | BGC0000136 | Rifamycin\_biosynthetic\_gene\_cluster | 33 | 34.5284489478 | 398 | 8.1e-110 |
| AAZ77696.1 | ChlA3 | BGC0000036 | Chlorothricin\_biosynthetic\_gene\_cluster | 32 | 35.7365549493 | 397 | 1.1e-109 |
| ADM46358.1 | polyketide\_synthase | BGC0000106 | Naphthomycin\_biosynthetic\_gene\_cluster | 34 | 35.2299298519 | 397 | 1.1e-109 |
| AGC45623.1 | polyketide\_synthase | BGC0001394 | Phenalamide\_biosynthetic\_gene\_cluster | 33 | 35.0740452065 | 397 | 1.1e-109 |
| AGC45621.1 | polyketide\_synthase | BGC0001394 | Phenalamide\_biosynthetic\_gene\_cluster | 30 | 40.8028059236 | 397 | 1.1e-109 |
| ABK32291.1 | JerE | BGC0000080 | Jerangolid\_biosynthetic\_gene\_cluster | 31 | 37.8020265004 | 397 | 1.1e-109 |
| AAP42858.1 | NanA4 | BGC0000105 | Nanchangmycin\_biosynthetic\_gene\_cluster | 33 | 36.5159781761 | 397 | 1.4e-109 |
| BAB69196.1 | modular\_polyketide\_synthase | BGC0000117 | Oligomycin\_biosynthetic\_gene\_cluster | 31 | 39.4388152767 | 396 | 1.8e-109 |
| CAQ43075.1 | polyketide\_synthase | BGC0000970 | Chondrochloren\_biosynthetic\_gene\_cluster | 30 | 45.2065471551 | 396 | 1.8e-109 |
| AAK73503.1 | AmphK | BGC0000015 | Amphotericin\_biosynthetic\_gene\_cluster | 32 | 35.2299298519 | 396 | 1.8e-109 |
| ADC79637.1 | TamAI | BGC0001052 | Tirandamycin\_biosynthetic\_gene\_cluster | 31 | 35.0350740452 | 396 | 1.8e-109 |
| AHH34189.1 | polyketide\_synthase | BGC0001162 | 1-heptadecene\_biosynthetic\_gene\_cluster | 31 | 37.8409976617 | 396 | 1.8e-109 |
| ABC87509.1 | polyketide\_synthase | BGC0001011 | Meridamycin\_biosynthetic\_gene\_cluster | 33 | 37.0226032736 | 396 | 1.8e-109 |
| CCP20051.1 | divM\_protein | BGC0001119 | Divergolide\_biosynthetic\_gene\_cluster | 31 | 44.8168355417 | 396 | 2.3e-109 |
| AKD43768.1 | HerA1 | BGC0001349 | Heronamide\_biosynthetic\_gene\_cluster | 33 | 35.1909586906 | 396 | 2.3e-109 |
| ACB46197.1 | polyketide\_synthase | BGC0000989 | Epothilone\_biosynthetic\_gene\_cluster | 31 | 35.2299298519 | 396 | 2.3e-109 |
| AAG23264.1 | polyketide\_synthase\_loading\_and\_extender\_module\_1 | BGC0000148 | Spinosad\_biosynthetic\_gene\_cluster | 32 | 37.1005455963 | 396 | 3.1e-109 |
| AEZ54374.1 | PieA1 | BGC0000124 | Piericidin\_A1\_biosynthetic\_gene\_cluster | 31 | 39.477786438 | 396 | 3.1e-109 |
| CAO98850.1 | polyketide\_synthase\_AufG | BGC0000023 | Aurafuron\_biosynthetic\_gene\_cluster | 31 | 35.0740452065 | 395 | 4e-109 |
| ctg1\_orf20 |  | BGC0001013 | Meridamycin\_biosynthetic\_gene\_cluster | 33 | 36.2431800468 | 395 | 4e-109 |
| AAF26919.1 | polyketide\_synthase | BGC0000988 | Epothilone\_biosynthetic\_gene\_cluster | 32 | 35.3078721746 | 395 | 4e-109 |
| ADC45534.1 | modular\_polyketide\_synthase | BGC0000093 | Meilingmycin\_biosynthetic\_gene\_cluster | 30 | 40.3351519875 | 395 | 5.2e-109 |
| ANI24099.1 | polyketide\_synthase | BGC0001235 | Antalid\_biosynthetic\_gene\_cluster | 32 | 36.1652377241 | 395 | 5.2e-109 |
| BAF02923.1 | type\_I\_polyketide\_synthase | BGC0000073 | Halstoctacosanolide\_biosynthetic\_gene\_cluster | 31 | 39.4388152767 | 395 | 5.2e-109 |
| AHA38203.1 | GphJ | BGC0000069 | Gephyronic\_acid\_biosynthetic\_gene\_cluster | 32 | 35.1519875292 | 395 | 5.2e-109 |
| BAE93729.1 | type\_I\_polyketide\_synthase | BGC0000164 | Tetronomycin\_biosynthetic\_gene\_cluster | 33 | 36.0872954014 | 394 | 6.8e-109 |
| ACR50785.1 | polyketide\_synthase | BGC0000163 | Tetronasin\_biosynthetic\_gene\_cluster | 30 | 44.505066251 | 394 | 6.8e-109 |
| BAF85843.1 | modular\_polyketide\_synthase | BGC0000109 | Nemadectin\_biosynthetic\_gene\_cluster | 32 | 35.46375682 | 394 | 6.8e-109 |
| CAD89775.1 | MelD\_protein | BGC0001009 | Melithiazol\_biosynthetic\_gene\_cluster | 28 | 50.5455962588 | 394 | 8.9e-109 |
| AAA79984.2 | soraphen\_polyketide\_synthase\_B | BGC0000147 | Soraphen\_biosynthetic\_gene\_cluster | 32 | 35.7755261107 | 394 | 8.9e-109 |
| BAC68126.1 | modular\_polyketide\_synthase | BGC0000059 | Filipin\_biosynthetic\_gene\_cluster | 32 | 36.3990646921 | 394 | 8.9e-109 |
| ctg1\_orf524 |  | BGC0001199 | Akaeolide\_biosynthetic\_gene\_cluster | 33 | 35.0350740452 | 394 | 8.9e-109 |
| CBD77732.1 | polyketide\_synthase | BGC0000974 | Crocacin\_biosynthetic\_gene\_cluster | 32 | 37.0615744349 | 394 | 8.9e-109 |
| CCE88379.1 | polyketide\_synthase | BGC0001034 | Pellasoren\_biosynthetic\_gene\_cluster | 29 | 47.9734996103 | 394 | 1.2e-108 |
| ctg1\_orf521 |  | BGC0001199 | Akaeolide\_biosynthetic\_gene\_cluster | 33 | 35.3078721746 | 394 | 1.2e-108 |
| BAE93722.1 | type\_I\_polyketide\_synthase | BGC0000164 | Tetronomycin\_biosynthetic\_gene\_cluster | 32 | 35.9703819174 | 394 | 1.2e-108 |
| ABV91286.1 | type\_I\_modular\_polyketide\_synthase | BGC0000158 | Tautomycetin\_biosynthetic\_gene\_cluster | 32 | 35.7365549493 | 394 | 1.2e-108 |
| AAC46024.1 | polyketide\_synthase\_modules\_1\_and\_2 | BGC0000113 | Niddamycin\_biosynthetic\_gene\_cluster | 31 | 34.7622759158 | 394 | 1.2e-108 |
| AAF19810.1 | MtaB | BGC0001024 | Myxothiazol\_biosynthetic\_gene\_cluster | 32 | 36.8277474669 | 393 | 1.5e-108 |
| ALA09357.1 | type\_I\_modular\_PKS | BGC0001303 | Lobosamide\_biosynthetic\_gene\_cluster | 33 | 35.1130163679 | 393 | 1.5e-108 |
| AFV30247.1 | polyketide\_synthase | BGC0000075 | Hygrocin\_biosynthetic\_gene\_cluster | 31 | 39.24395947 | 393 | 1.5e-108 |
| CAQ18834.1 | polyketide\_synthase | BGC0000954 | Ajudazol\_biosynthetic\_gene\_cluster | 32 | 35.0350740452 | 393 | 1.5e-108 |
| AAF26923.1 | polyketide\_synthase | BGC0000988 | Epothilone\_biosynthetic\_gene\_cluster | 31 | 35.2689010133 | 393 | 2e-108 |
| ABK32288.1 | JerB | BGC0000080 | Jerangolid\_biosynthetic\_gene\_cluster | 31 | 40.1792673422 | 393 | 2.6e-108 |
| AEH42491.1 | polyketide\_synthase | BGC0000032 | Calcimycin\_biosynthetic\_gene\_cluster | 31 | 38.3476227592 | 393 | 2.6e-108 |
| ADH04657.1 | TugA | BGC0001342 | Thuggacin\_biosynthetic\_gene\_cluster | 30 | 39.9844115355 | 393 | 2.6e-108 |
| AAO62582.1 | polyketide\_synthase\_peptide\_sythetase\_fusion\_protein | BGC0001016 | Microcystin\_biosynthetic\_gene\_cluster | 33 | 36.1262665627 | 392 | 3.4e-108 |
| ABK32289.1 | JerC | BGC0000080 | Jerangolid\_biosynthetic\_gene\_cluster | 32 | 38.8152766952 | 392 | 3.4e-108 |
| ABL86391.1 | hybrid\_polyketide\_synthase\_and\_nonribosomal\_peptide\_synthetase | BGC0000999 | Heat-stable\_antifungal\_factor\_biosynthetic\_gene\_cluster | 30 | 43.8815276695 | 392 | 3.4e-108 |
| AAW03327.1 | CtaD | BGC0000982 | Cystothiazole\_A\_biosynthetic\_gene\_cluster | 29 | 50.233826968 | 392 | 4.4e-108 |
| AAF86393.1 | FkbB | BGC0000994 | FK520\_biosynthetic\_gene\_cluster | 31 | 40.4130943102 | 392 | 4.4e-108 |
| CAC20931.1 | PimS1\_protein | BGC0000125 | Pimaricin\_biosynthetic\_gene\_cluster | 31 | 37.1005455963 | 392 | 4.4e-108 |
| ADH04658.1 | TugB | BGC0001342 | Thuggacin\_biosynthetic\_gene\_cluster | 32 | 38.3086515978 | 392 | 4.4e-108 |
| AAP42859.1 | NanA5 | BGC0000105 | Nanchangmycin\_biosynthetic\_gene\_cluster | 31 | 39.9844115355 | 391 | 5.8e-108 |
| ACO94484.1 | polyketide\_synthase\_type\_I | BGC0000097 | ML-449\_biosynthetic\_gene\_cluster | 31 | 38.2307092751 | 391 | 5.8e-108 |
| AEZ54379.1 | PieA6 | BGC0000124 | Piericidin\_A1\_biosynthetic\_gene\_cluster | 30 | 38.1527669525 | 391 | 5.8e-108 |
| BAF02921.1 | type\_I\_polyketide\_synthase | BGC0000073 | Halstoctacosanolide\_biosynthetic\_gene\_cluster | 33 | 35.8924395947 | 391 | 5.8e-108 |
| AAF62880.1 | EpoA | BGC0000991 | Epothilone\_biosynthetic\_gene\_cluster | 32 | 35.1909586906 | 391 | 7.6e-108 |
| ABJ97437.1 | MerA | BGC0001012 | Meridamycin\_biosynthetic\_gene\_cluster | 33 | 36.7108339829 | 391 | 7.6e-108 |
| AAF62885.1 | EpoF | BGC0000991 | Epothilone\_biosynthetic\_gene\_cluster | 31 | 35.2299298519 | 391 | 7.6e-108 |
| ABK32256.1 | AmbB | BGC0000014 | Ambruticin\_biosynthetic\_gene\_cluster | 32 | 35.1909586906 | 391 | 7.6e-108 |
| ABC84459.1 | NigAIV | BGC0000114 | Nigericin\_biosynthetic\_gene\_cluster | 32 | 36.2042088854 | 391 | 9.9e-108 |
| ACB37741.1 | putative\_type\_I\_polyketide\_synthase | BGC0000162 | Tetrocarcin\_A\_biosynthetic\_gene\_cluster | 33 | 34.8402182385 | 391 | 9.9e-108 |
| ABK32257.1 | AmbC | BGC0000014 | Ambruticin\_biosynthetic\_gene\_cluster | 32 | 35.580670304 | 390 | 1.3e-107 |
| CAD17792.1 | probable\_non\_ribosomal\_peptide\_synthetase\_protein | BGC0001363 | Ralsolamycin\_biosynthetic\_gene\_cluster | 31 | 36.5549493375 | 390 | 1.3e-107 |
| AFP87523.1 | type\_I\_polyketide\_synthase | BGC0001159 | Marinopyrrole\_biosynthetic\_gene\_cluster | 32 | 36.5549493375 | 390 | 1.3e-107 |
| ABO15861.1 | polyketide\_synthase | BGC0000130 | Pyrrolomycin\_biosynthetic\_gene\_cluster | 32 | 35.580670304 | 390 | 1.7e-107 |
| BAD08359.1 | polyketide\_synthase\_modules\_5-6 | BGC0000167 | Vicenistatin\_biosynthetic\_gene\_cluster | 33 | 35.1519875292 | 389 | 2.2e-107 |
| BAF85839.1 | modular\_polyketide\_synthase | BGC0000109 | Nemadectin\_biosynthetic\_gene\_cluster | 31 | 35.580670304 | 389 | 2.2e-107 |
| AFL48527.1 | laidlomycin\_polyketide\_synthase\_(module\_3\_and\_module\_4) | BGC0000084 | Laidlomycin\_biosynthetic\_gene\_cluster | 30 | 40.6079501169 | 389 | 2.9e-107 |
| ADB12488.1 | EpoA | BGC0000990 | Epothilone\_biosynthetic\_gene\_cluster | 32 | 35.1909586906 | 389 | 2.9e-107 |
| ctg1\_orf10 |  | BGC0000053 | Elaiophylin\_biosynthetic\_gene\_cluster | 29 | 43.8425565082 | 389 | 2.9e-107 |
| ACB46192.1 | polyketide\_synthase | BGC0000989 | Epothilone\_biosynthetic\_gene\_cluster | 32 | 35.1909586906 | 389 | 2.9e-107 |
| ADB12493.1 | EpoF | BGC0000990 | Epothilone\_biosynthetic\_gene\_cluster | 31 | 35.2299298519 | 389 | 2.9e-107 |
| AAX98186.1 | polyketide\_synthase\_type\_I | BGC0000052 | ECO-02301\_biosynthetic\_gene\_cluster | 31 | 37.646141855 | 389 | 2.9e-107 |
| ADX66472.1 | ScnS1 | BGC0000108 | Natamycin\_biosynthetic\_gene\_cluster | 31 | 37.4902572097 | 389 | 2.9e-107 |
| AEZ53947.1 | polyketide\_synthase | BGC0000144 | Salinomycin\_biosynthetic\_gene\_cluster | 33 | 36.0093530787 | 389 | 3.7e-107 |
| ADC45538.1 | modular\_polyketide\_synthase | BGC0000093 | Meilingmycin\_biosynthetic\_gene\_cluster | 31 | 35.5027279813 | 389 | 3.7e-107 |
| AAQ82564.1 | FscC | BGC0000034 | Candicidin\_biosynthetic\_gene\_cluster | 32 | 35.0740452065 | 388 | 4.9e-107 |
| AAN32979.1 | BarE | BGC0000962 | Barbamide\_biosynthetic\_gene\_cluster | 31 | 37.5681995323 | 388 | 4.9e-107 |
| AFL48525.1 | laidlomycin\_polyketide\_synthase\_(loading\_module\_and\_module\_1) | BGC0000084 | Laidlomycin\_biosynthetic\_gene\_cluster | 33 | 35.580670304 | 388 | 4.9e-107 |
| AAX98185.1 | polyketide\_synthase\_type\_I | BGC0000052 | ECO-02301\_biosynthetic\_gene\_cluster | 31 | 34.8402182385 | 388 | 4.9e-107 |
| ADM46359.1 | polyketide\_synthase | BGC0000106 | Naphthomycin\_biosynthetic\_gene\_cluster | 33 | 35.3858144973 | 388 | 6.4e-107 |
| BAG17643.1 | putative\_NRPS-type-I\_PKS\_fusion\_protein | BGC0001043 | SGR\_PTMs\_biosynthetic\_gene\_cluster | 30 | 45.2844894778 | 388 | 6.4e-107 |
| AFL48526.1 | laidlomycin\_polyketide\_synthase\_(module\_2) | BGC0000084 | Laidlomycin\_biosynthetic\_gene\_cluster | 31 | 35.2299298519 | 388 | 8.4e-107 |
| EGJ35088.1 | polyketide\_synthase\_module | BGC0001163 | 1-heptadecene\_biosynthetic\_gene\_cluster | 31 | 37.1005455963 | 388 | 8.4e-107 |
| CAJ88175.1 | putative\_polyketide\_synthase\_B | BGC0000151 | Stambomycin\_biosynthetic\_gene\_cluster | 33 | 35.6586126267 | 387 | 1.1e-106 |
| AAQ90174.1 | polyketide\_synthase\_type\_I | BGC0000128 | Pyoluteorin\_biosynthetic\_gene\_cluster | 32 | 35.3468433359 | 387 | 1.4e-106 |
| ADC79638.1 | TamAII | BGC0001052 | Tirandamycin\_biosynthetic\_gene\_cluster | 30 | 35.2689010133 | 387 | 1.4e-106 |
| CAJ88176.1 | putative\_polyketide\_synthase\_B | BGC0000151 | Stambomycin\_biosynthetic\_gene\_cluster | 30 | 44.4660950896 | 387 | 1.4e-106 |
| ACY06288.1 | type\_I\_polyketide\_synthase | BGC0001042 | Sanglifehrin\_A\_biosynthetic\_gene\_cluster | 30 | 41.7770849571 | 386 | 1.9e-106 |
| AAC69329.1 | type\_I\_polyketide\_synthase\_PikAI | BGC0000094 | Methymycin\_/\_pikromycin\_biosynthetic\_gene\_cluster | 31 | 36.9446609509 | 386 | 1.9e-106 |
| ctg1\_orf11 |  | BGC0000053 | Elaiophylin\_biosynthetic\_gene\_cluster | 30 | 39.360872954 | 386 | 1.9e-106 |
| AAF71774.1 | NysA | BGC0000115 | Nystatin\_biosynthetic\_gene\_cluster | 32 | 34.9961028839 | 386 | 2.4e-106 |
| AHB82052.1 | polyketide\_synthase | BGC0001019 | Microsclerodermins\_biosynthetic\_gene\_cluster | 37 | 25.4091971941 | 386 | 2.4e-106 |
| AAS92537.1 | PKS1 | BGC0001044 | Sirodesmin\_biosynthetic\_gene\_cluster | 31 | 35.5027279813 | 386 | 2.4e-106 |
| AAQ82568.1 | FscD | BGC0000061 | FR-008\_biosynthetic\_gene\_cluster | 33 | 35.8924395947 | 386 | 2.4e-106 |
| CAQ34920.1 | polyketide\_synthase | BGC0000986 | Dkxanthene\_biosynthetic\_gene\_cluster | 30 | 38.9711613406 | 386 | 2.4e-106 |
| AAO65806.1 | monensin\_polyketide\_synthase\_modules\_11\_and\_12 | BGC0000100 | Monensin\_biosynthetic\_gene\_cluster | 30 | 44.5830085737 | 386 | 2.4e-106 |
| CAA16183.1 | polyketide\_synthase | BGC0001063 | Undecylprodigiosin\_biosynthetic\_gene\_cluster | 34 | 31.9563522993 | 386 | 2.4e-106 |
| BAC68125.1 | modular\_polyketide\_synthase | BGC0000059 | Filipin\_biosynthetic\_gene\_cluster | 32 | 35.3858144973 | 386 | 3.2e-106 |
| ABB88522.1 | polyketide\_synthase\_type\_I | BGC0000050 | E-837\_biosynthetic\_gene\_cluster | 32 | 34.9571317225 | 386 | 3.2e-106 |
| CAQ34918.1 | nonribosomal\_peptide\_synthetase/\_polyketide\_synthase | BGC0000986 | Dkxanthene\_biosynthetic\_gene\_cluster | 28 | 48.1293842557 | 385 | 4.1e-106 |
| BAG85032.1 | putative\_polyketide\_synthase | BGC0000086 | Lasalocid\_biosynthetic\_gene\_cluster | 33 | 34.5674201091 | 385 | 4.1e-106 |
| AJO72736.1 | Type\_I\_modular\_polyketide\_synthase | BGC0001381 | Brasilinolide\_biosynthetic\_gene\_cluster | 34 | 32.6578332034 | 385 | 5.4e-106 |
| CAQ43077.1 | polyketide\_synthase | BGC0000970 | Chondrochloren\_biosynthetic\_gene\_cluster | 28 | 50.7014809041 | 385 | 5.4e-106 |
| CBD77734.1 | polyketide\_synthase | BGC0000974 | Crocacin\_biosynthetic\_gene\_cluster | 31 | 35.5027279813 | 384 | 7.1e-106 |
| AFI57005.1 | QmnA1 | BGC0000133 | Quartromicin\_biosynthetic\_gene\_cluster | 32 | 34.6843335931 | 384 | 7.1e-106 |
| AEZ54378.1 | PieA5 | BGC0000124 | Piericidin\_A1\_biosynthetic\_gene\_cluster | 31 | 39.9454403741 | 384 | 7.1e-106 |
| AFU82617.1 | polyketide\_synthase | BGC0000998 | Guadinomine\_biosynthetic\_gene\_cluster | 32 | 35.1909586906 | 384 | 9.2e-106 |
| CBA11582.1 | polyketide\_synthase\_type\_I | BGC0001046 | Streptolydigin\_biosynthetic\_gene\_cluster | 31 | 36.0483242401 | 384 | 9.2e-106 |
| CAL58685.1 | polyketide\_synthase | BGC0000149 | Spirangien\_biosynthetic\_gene\_cluster | 32 | 35.3858144973 | 384 | 9.2e-106 |
| ctg1\_orf7 |  | BGC0000053 | Elaiophylin\_biosynthetic\_gene\_cluster | 32 | 34.9181605612 | 384 | 9.2e-106 |
| BAF02922.1 | type\_I\_polyketide\_synthase | BGC0000073 | Halstoctacosanolide\_biosynthetic\_gene\_cluster | 32 | 36.0093530787 | 384 | 1.2e-105 |
| BAD08360.1 | polyketide\_synthase\_modules\_7-8 | BGC0000167 | Vicenistatin\_biosynthetic\_gene\_cluster | 31 | 40.5689789556 | 384 | 1.2e-105 |
| AEK75502.1 | type\_1\_polyketide\_synthase | BGC0000001 | Abyssomicin\_biosynthetic\_gene\_cluster | 32 | 35.1130163679 | 384 | 1.2e-105 |
| WP\_043476519.1 | polyketide\_synthase | BGC0001232 | Leucanicidin\_biosynthetic\_gene\_cluster | 32 | 41.5432579891 | 384 | 1.2e-105 |
| AKD43764.1 | HerF | BGC0001349 | Heronamide\_biosynthetic\_gene\_cluster | 31 | 38.9711613406 | 384 | 1.2e-105 |
| AIT55263.1 | GulF | BGC0000072 | Gulmirecin\_biosynthetic\_gene\_cluster | 28 | 46.0249415433 | 383 | 1.6e-105 |
| ADH04641.1 | TgaC | BGC0001051 | Thuggacin\_biosynthetic\_gene\_cluster | 33 | 35.7365549493 | 383 | 1.6e-105 |
| CAJ88185.2 | putative\_modular\_polyketide\_synthase | BGC0000151 | Stambomycin\_biosynthetic\_gene\_cluster | 32 | 35.2299298519 | 383 | 1.6e-105 |
| BAF02924.1 | type\_I\_polyketide\_synthase | BGC0000073 | Halstoctacosanolide\_biosynthetic\_gene\_cluster | 30 | 39.5557287607 | 383 | 2.1e-105 |
| AEZ53946.1 | polyketide\_synthase | BGC0000144 | Salinomycin\_biosynthetic\_gene\_cluster | 33 | 35.6196414653 | 383 | 2.1e-105 |
| AAQ84157.1 | Plm2-3 | BGC0000123 | Phoslactomycin\_B\_biosynthetic\_gene\_cluster | 31 | 37.0226032736 | 383 | 2.1e-105 |
| ADC45586.1 | modular\_polyketide\_synthase | BGC0000093 | Meilingmycin\_biosynthetic\_gene\_cluster | 29 | 48.5970381917 | 383 | 2.1e-105 |
| AJW65409.1 | type\_I\_modular\_polyketide\_synthase | BGC0001195 | Nocardiopsin\_biosynthetic\_gene\_cluster | 32 | 36.3211223694 | 383 | 2.7e-105 |
| ALA09371.1 | type\_I\_modular\_PKS | BGC0001303 | Lobosamide\_biosynthetic\_gene\_cluster | 32 | 35.1130163679 | 383 | 2.7e-105 |
| ACR50775.1 | polyketide\_synthase | BGC0000163 | Tetronasin\_biosynthetic\_gene\_cluster | 31 | 39.9844115355 | 383 | 2.7e-105 |
| ADC79639.1 | TamAIII | BGC0001052 | Tirandamycin\_biosynthetic\_gene\_cluster | 31 | 45.0896336711 | 383 | 2.7e-105 |
| AAO65797.1 | monensin\_polyketide\_synthase\_module\_2 | BGC0000100 | Monensin\_biosynthetic\_gene\_cluster | 31 | 34.9571317225 | 383 | 2.7e-105 |
| AJO72743.1 | Type\_I\_modular\_polyketide\_synthase | BGC0001381 | Brasilinolide\_biosynthetic\_gene\_cluster | 31 | 34.6063912705 | 382 | 3.5e-105 |
| CUW01171.1 | Type\_I\_polyketide\_synthase | BGC0001359 | PM100117\_/\_PM100118\_biosynthetic\_gene\_cluster | 30 | 35.4247856586 | 382 | 4.6e-105 |
| BAE93730.1 | type\_I\_polyketide\_synthase | BGC0000164 | Tetronomycin\_biosynthetic\_gene\_cluster | 30 | 39.7116134061 | 382 | 4.6e-105 |
| ACN69989.1 | polyketide\_synthase | BGC0000079 | Indanomycin\_biosynthetic\_gene\_cluster | 30 | 41.8939984412 | 382 | 4.6e-105 |
| CAD55506.1 | putative\_type\_I\_polyketide\_synthase | BGC0000038 | Coelimycin\_biosynthetic\_gene\_cluster | 32 | 35.931410756 | 382 | 4.6e-105 |
| AAX98189.1 | polyketide\_synthase\_type\_I | BGC0000052 | ECO-02301\_biosynthetic\_gene\_cluster | 31 | 39.7505845674 | 381 | 6e-105 |
| AAG23263.1 | polyketide\_synthase\_extender\_modules\_5-7 | BGC0000148 | Spinosad\_biosynthetic\_gene\_cluster | 32 | 35.814497272 | 381 | 6e-105 |
| BAH02268.1 | polyketide\_synthase | BGC0000126 | Pladienolide\_biosynthetic\_gene\_cluster | 31 | 35.0350740452 | 381 | 6e-105 |
| ABM21569.1 | CrpA | BGC0000975 | Cryptophycin\_biosynthetic\_gene\_cluster | 30 | 36.4380358535 | 381 | 6e-105 |
| AHN85651.1 | Phn2 | BGC0000122 | Phenylnannolone\_A\_biosynthetic\_gene\_cluster | 30 | 39.0880748246 | 381 | 7.8e-105 |
| AFU82614.1 | mixed\_NRPS\_PKS | BGC0000998 | Guadinomine\_biosynthetic\_gene\_cluster | 30 | 40.958690569 | 381 | 7.8e-105 |
| CBZ41585.1 | putative\_modular\_polyketide\_synthase | BGC0000151 | Stambomycin\_biosynthetic\_gene\_cluster | 32 | 38.1527669525 | 381 | 7.8e-105 |
| AHF22854.1 | MarL | BGC0000091 | Marineosin\_biosynthetic\_gene\_cluster | 33 | 32.385035074 | 381 | 7.8e-105 |
| AEH42490.1 | polyketide\_synthase | BGC0000032 | Calcimycin\_biosynthetic\_gene\_cluster | 31 | 37.1784879189 | 381 | 7.8e-105 |
| CAC20920.1 | PimS3\_protein | BGC0000125 | Pimaricin\_biosynthetic\_gene\_cluster | 34 | 34.6453624318 | 381 | 1e-104 |
| ALP32042.1 | CycB | BGC0001293 | Cyclizidine\_biosynthetic\_gene\_cluster | 31 | 35.3858144973 | 381 | 1e-104 |
| AAQ82567.1 | FscE | BGC0000061 | FR-008\_biosynthetic\_gene\_cluster | 32 | 35.6586126267 | 381 | 1e-104 |
| ABP55210.1 | beta-ketoacyl\_synthase | BGC0000142 | Salinilactam\_biosynthetic\_gene\_cluster | 30 | 45.1675759938 | 381 | 1e-104 |
| BAJ16470.1 | polyketide\_synthase | BGC0000058 | FD-891\_biosynthetic\_gene\_cluster | 30 | 39.4388152767 | 381 | 1e-104 |
| CAA60462.1 | polyketide\_synthase | BGC0001040 | Rapamycin\_biosynthetic\_gene\_cluster | 31 | 39.2829306313 | 380 | 1.7e-104 |
| CAD15508.1 | probable\_polyketide\_synthase\_protein | BGC0001014 | Micacocidin\_biosynthetic\_gene\_cluster | 33 | 36.7108339829 | 380 | 1.7e-104 |
| AAX98184.1 | polyketide\_synthase\_type\_I | BGC0000052 | ECO-02301\_biosynthetic\_gene\_cluster | 32 | 35.4247856586 | 380 | 1.7e-104 |
| AAO65801.1 | monensin\_polyketide\_synthase\_module\_9 | BGC0000100 | Monensin\_biosynthetic\_gene\_cluster | 33 | 36.0872954014 | 380 | 1.7e-104 |
| ABB52545.1 | putative\_type\_I\_polyketide\_synthase | BGC0000047 | Dihydrochalcomycin\_biosynthetic\_gene\_cluster | 32 | 36.3211223694 | 379 | 2.3e-104 |
| ANC94962.1 | AlmHV | BGC0001396 | Aldgamycin\_biosynthetic\_gene\_cluster | 31 | 36.2821512081 | 379 | 2.3e-104 |
| BAD08373.1 | polyketide\_synthase\_modules\_1-3 | BGC0000167 | Vicenistatin\_biosynthetic\_gene\_cluster | 31 | 39.0491036633 | 379 | 2.3e-104 |
| AAG23266.1 | polyketide\_synthase\_extender\_modules\_3-4 | BGC0000148 | Spinosad\_biosynthetic\_gene\_cluster | 32 | 36.0483242401 | 379 | 2.3e-104 |
| ADH04640.1 | TgaB | BGC0001051 | Thuggacin\_biosynthetic\_gene\_cluster | 32 | 35.3078721746 | 379 | 2.3e-104 |
| ABK32263.1 | AmbH | BGC0000014 | Ambruticin\_biosynthetic\_gene\_cluster | 32 | 35.1519875292 | 379 | 3.9e-104 |
| ABX60161.1 | mixed\_NRPS/PKS | BGC0000978 | Cylindrospermopsin\_biosynthetic\_gene\_cluster | 29 | 41.8939984412 | 379 | 3.9e-104 |
| BAH02270.1 | polyketide\_synthase | BGC0000126 | Pladienolide\_biosynthetic\_gene\_cluster | 29 | 46.9212782541 | 379 | 3.9e-104 |
| AAS90093.1 | PksA | BGC0000006 | Aflatoxin\_biosynthetic\_gene\_cluster | 30 | 35.2689010133 | 379 | 3.9e-104 |
| CAC20919.1 | PimS4\_protein | BGC0000125 | Pimaricin\_biosynthetic\_gene\_cluster | 33 | 35.1519875292 | 378 | 5.1e-104 |
| BAO66519.1 | type\_I\_polyketide\_synthase | BGC0000042 | Cremimycin\_biosynthetic\_gene\_cluster | 30 | 43.4528448948 | 378 | 5.1e-104 |
| ctg1\_orf522 |  | BGC0001199 | Akaeolide\_biosynthetic\_gene\_cluster | 29 | 44.1932969602 | 378 | 5.1e-104 |
| CAL58683.1 | polyketide\_synthase | BGC0000149 | Spirangien\_biosynthetic\_gene\_cluster | 31 | 37.4902572097 | 378 | 5.1e-104 |
| CAL58681.1 | polyketide\_synthase | BGC0000149 | Spirangien\_biosynthetic\_gene\_cluster | 32 | 32.3460639127 | 378 | 5.1e-104 |
| AAS89999.1 | PksA | BGC0000007 | Aflatoxin\_biosynthetic\_gene\_cluster | 31 | 35.1130163679 | 378 | 5.1e-104 |
| ctg1\_orf254 |  | BGC0001200 | Lorneic\_acid\_A\_biosynthetic\_gene\_cluster | 30 | 43.6477007015 | 378 | 5.1e-104 |
| AEZ53950.1 | polyketide\_synthase | BGC0000144 | Salinomycin\_biosynthetic\_gene\_cluster | 32 | 35.6586126267 | 378 | 6.6e-104 |
| AGZ15474.1 | putative\_type\_I\_polyketide\_synthase | BGC0001036 | Polyoxypeptin\_biosynthetic\_gene\_cluster | 31 | 39.7116134061 | 378 | 6.6e-104 |
| CBA11584.1 | polyketide\_synthase\_type\_I | BGC0001046 | Streptolydigin\_biosynthetic\_gene\_cluster | 31 | 35.46375682 | 378 | 6.6e-104 |
| ABK32255.1 | AmbA | BGC0000014 | Ambruticin\_biosynthetic\_gene\_cluster | 33 | 35.1909586906 | 378 | 6.6e-104 |
| AJW65408.1 | type\_I\_modular\_polyketide\_synthase | BGC0001195 | Nocardiopsin\_biosynthetic\_gene\_cluster | 32 | 35.5416991426 | 378 | 8.6e-104 |
| ABV97151.1 | AMP-dependent\_synthetase\_and\_ligase | BGC0000137 | Rifamycin\_biosynthetic\_gene\_cluster | 31 | 35.580670304 | 378 | 8.6e-104 |
| BAQ25511.1 | type\_I\_polyketide\_synthase | BGC0001288 | Maklamicin\_biosynthetic\_gene\_cluster | 32 | 34.8791893998 | 378 | 8.6e-104 |
| ALP32046.1 | CycF | BGC0001293 | Cyclizidine\_biosynthetic\_gene\_cluster | 29 | 44.5440374123 | 378 | 8.6e-104 |
| AAO65799.1 | monensin\_polyketide\_synthase\_modules\_5\_and\_6 | BGC0000100 | Monensin\_biosynthetic\_gene\_cluster | 32 | 35.697583788 | 378 | 8.6e-104 |
| AHB82070.1 | polyketide\_synthase | BGC0001231 | Microsclerodermins\_biosynthetic\_gene\_cluster | 31 | 35.697583788 | 377 | 1.1e-103 |
| AHH99922.1 | PKS\_I | BGC0000002 | Aculeximycin\_biosynthetic\_gene\_cluster | 32 | 34.9961028839 | 377 | 1.5e-103 |
| AAK73501.1 | AmphI | BGC0000015 | Amphotericin\_biosynthetic\_gene\_cluster | 31 | 35.0740452065 | 377 | 1.5e-103 |
| BAF85838.1 | modular\_polyketide\_synthase | BGC0000109 | Nemadectin\_biosynthetic\_gene\_cluster | 31 | 36.3600935308 | 377 | 1.5e-103 |
| BAR73021.1 | putative\_PKS\_(KS-AT-KR-ACP) | BGC0001194 | Hitachimycin\_biosynthetic\_gene\_cluster | 31 | 35.0740452065 | 377 | 1.5e-103 |
| AJW65407.1 | type\_I\_modular\_polyketide\_synthase | BGC0001195 | Nocardiopsin\_biosynthetic\_gene\_cluster | 32 | 35.5416991426 | 376 | 1.9e-103 |
| CAA60460.1 | polyketide\_synthase | BGC0001040 | Rapamycin\_biosynthetic\_gene\_cluster | 32 | 35.4247856586 | 376 | 1.9e-103 |
| BAE71314.1 | polyketide\_synthase | BGC0000004 | Aflatoxin\_biosynthetic\_gene\_cluster | 30 | 35.2689010133 | 376 | 1.9e-103 |
| AGY30677.1 | Ann5 | BGC0001298 | 4-Z-annimycin\_biosynthetic\_gene\_cluster | 31 | 36.0483242401 | 376 | 1.9e-103 |
| CAM00064.1 | EryAII\_Erythromycin\_polyketide\_synthase\_modules\_3\_and\_4 | BGC0000055 | Erythromycin\_biosynthetic\_gene\_cluster | 31 | 35.2689010133 | 376 | 2.5e-103 |
| BAG85027.1 | putative\_polyketide\_synthase | BGC0000086 | Lasalocid\_biosynthetic\_gene\_cluster | 29 | 44.0763834762 | 376 | 2.5e-103 |
| AEZ64503.1 | Herd | BGC0001065 | Herboxidiene\_biosynthetic\_gene\_cluster | 31 | 34.9961028839 | 376 | 2.5e-103 |
| ADF88279.1 | mixed\_NRPS/PKS | BGC0000981 | Cylindrospermopsin\_biosynthetic\_gene\_cluster | 29 | 41.9329696025 | 376 | 2.5e-103 |
| CAQ64687.1 | lasalocid\_modular\_polyketide\_synthase | BGC0000087 | Lasalocid\_biosynthetic\_gene\_cluster | 29 | 44.0763834762 | 376 | 2.5e-103 |
| AAQ82566.1 | FscF | BGC0000034 | Candicidin\_biosynthetic\_gene\_cluster | 32 | 35.2299298519 | 376 | 3.3e-103 |
| AEZ54377.1 | PieA4 | BGC0000124 | Piericidin\_A1\_biosynthetic\_gene\_cluster | 30 | 39.0491036633 | 376 | 3.3e-103 |
| BAB69194.1 | modular\_polyketide\_synthase | BGC0000117 | Oligomycin\_biosynthetic\_gene\_cluster | 32 | 35.697583788 | 375 | 4.3e-103 |
| ctg1\_orf256 |  | BGC0001200 | Lorneic\_acid\_A\_biosynthetic\_gene\_cluster | 30 | 44.3491816056 | 375 | 4.3e-103 |
| AAK73514.1 | AmphC | BGC0000015 | Amphotericin\_biosynthetic\_gene\_cluster | 32 | 34.6843335931 | 375 | 5.6e-103 |
| BAF02927.1 | type\_I\_polyketide\_synthase | BGC0000073 | Halstoctacosanolide\_biosynthetic\_gene\_cluster | 29 | 44.621979735 | 375 | 5.6e-103 |
| CAA60459.1 | polyketide\_synthase | BGC0001040 | Rapamycin\_biosynthetic\_gene\_cluster | 31 | 39.9454403741 | 375 | 5.6e-103 |
| AMK48247.1 | 6-methylsalicylic\_acid\_synthase | BGC0001351\_c2 | Cahuitamycins\_biosynthetic\_gene\_cluster | 34 | 31.2938425565 | 374 | 7.3e-103 |
| CAC22144.1 | putative\_type\_I\_polyketide\_synthase | BGC0000038 | Coelimycin\_biosynthetic\_gene\_cluster | 29 | 45.0896336711 | 374 | 7.3e-103 |
| ADC45535.1 | modular\_polyketide\_synthase | BGC0000093 | Meilingmycin\_biosynthetic\_gene\_cluster | 31 | 36.3600935308 | 374 | 7.3e-103 |
| ADA69241.1 | cis-AT\_polyketide\_synthase | BGC0001071 | Nosperin\_biosynthetic\_gene\_cluster | 30 | 35.5027279813 | 374 | 7.3e-103 |
| AEU17897.1 | putative\_type\_I\_PKS | BGC0001072 | Simocyclinone\_biosynthetic\_gene\_cluster | 29 | 39.9454403741 | 374 | 9.6e-103 |
| AIT55264.1 | GulA | BGC0000072 | Gulmirecin\_biosynthetic\_gene\_cluster | 33 | 32.1901792673 | 374 | 9.6e-103 |
| ADX66460.1 | ScnS3 | BGC0000108 | Natamycin\_biosynthetic\_gene\_cluster | 33 | 35.1130163679 | 374 | 1.2e-102 |
| AAS98784.1 | JamM | BGC0001001 | Jamaicamide\_biosynthetic\_gene\_cluster | 30 | 36.7498051442 | 374 | 1.2e-102 |
| AAZ77673.1 | ChlB1 | BGC0000036 | Chlorothricin\_biosynthetic\_gene\_cluster | 32 | 35.3468433359 | 373 | 1.6e-102 |
| CAD19085.1 | StiA\_protein | BGC0000153 | Stigmatellin\_biosynthetic\_gene\_cluster | 30 | 33.8269680436 | 373 | 1.6e-102 |
| AAS90022.1 | PksA | BGC0000008 | Aflatoxin\_biosynthetic\_gene\_cluster | 30 | 35.2689010133 | 373 | 1.6e-102 |
| ABV97152.1 | Beta-ketoacyl\_synthase | BGC0000137 | Rifamycin\_biosynthetic\_gene\_cluster | 29 | 44.3491816056 | 373 | 1.6e-102 |
| AAC69330.1 | type\_I\_polyketide\_synthase\_PikAII | BGC0000094 | Methymycin\_/\_pikromycin\_biosynthetic\_gene\_cluster | 29 | 45.5183164458 | 373 | 1.6e-102 |
| AAF86396.1 | FkbA | BGC0000994 | FK520\_biosynthetic\_gene\_cluster | 32 | 34.8402182385 | 373 | 1.6e-102 |
| BAQ25513.1 | type\_I\_polyketide\_synthase | BGC0001288 | Maklamicin\_biosynthetic\_gene\_cluster | 31 | 40.6079501169 | 373 | 1.6e-102 |
| ABV83222.1 | CppJ | BGC0000116 | Nystatin-like\_Pseudonocardia\_polyene\_biosynthetic\_gene\_cluster | 33 | 34.7622759158 | 373 | 1.6e-102 |
| ACY06286.1 | polyketide\_synthase | BGC0001042 | Sanglifehrin\_A\_biosynthetic\_gene\_cluster | 31 | 39.127045986 | 373 | 1.6e-102 |
| CAN89636.1 | putative\_polyketide\_synthase | BGC0001070 | Kirromycin\_biosynthetic\_gene\_cluster | 32 | 34.7622759158 | 373 | 2.1e-102 |
| AHH99924.1 | PKS\_I | BGC0000002 | Aculeximycin\_biosynthetic\_gene\_cluster | 30 | 39.3998441154 | 373 | 2.1e-102 |
| AAF71766.1 | NysI | BGC0000115 | Nystatin\_biosynthetic\_gene\_cluster | 31 | 35.1130163679 | 373 | 2.1e-102 |
| AEZ53948.1 | polyketide\_synthase | BGC0000144 | Salinomycin\_biosynthetic\_gene\_cluster | 32 | 35.580670304 | 373 | 2.1e-102 |
| BAG84248.1 | putative\_polyketide\_synthase | BGC0000257 | Prodigiosin\_biosynthetic\_gene\_cluster | 33 | 33.5931410756 | 373 | 2.1e-102 |
| ABB88520.1 | polyketide\_synthase\_type\_I | BGC0000050 | E-837\_biosynthetic\_gene\_cluster | 31 | 38.8152766952 | 373 | 2.1e-102 |
| ADX66461.1 | ScnS2 | BGC0000108 | Natamycin\_biosynthetic\_gene\_cluster | 31 | 35.580670304 | 373 | 2.8e-102 |
| ABI91466.1 | beta-ketoacyl\_synthase | BGC0001094 | Enacyloxin\_biosynthetic\_gene\_cluster | 32 | 35.3078721746 | 373 | 2.8e-102 |
| AAS90047.1 | PksA | BGC0000009 | Aflatoxin\_biosynthetic\_gene\_cluster | 30 | 35.1909586906 | 373 | 2.8e-102 |
| BAR73020.1 | putative\_PKS\_(KS-AT-DH-KR-ACP-KS-AT-DH-KR-ACP-KS-AT-DH-KR-ACP) | BGC0001194 | Hitachimycin\_biosynthetic\_gene\_cluster | 31 | 34.7622759158 | 372 | 3.6e-102 |
| AAS98787.1 | JamP | BGC0001001 | Jamaicamide\_biosynthetic\_gene\_cluster | 32 | 35.3858144973 | 372 | 3.6e-102 |
| ADC79618.1 | BafAIII | BGC0000028 | Bafilomycin\_biosynthetic\_gene\_cluster | 30 | 34.8012470772 | 372 | 3.6e-102 |
| AAP85336.1 | type\_I\_PKS | BGC0000233 | Hedamycin\_biosynthetic\_gene\_cluster | 31 | 35.6586126267 | 372 | 3.6e-102 |
| ACN69991.1 | polyketide\_synthase | BGC0000079 | Indanomycin\_biosynthetic\_gene\_cluster | 29 | 44.1543257989 | 372 | 4.7e-102 |
| AEZ54375.1 | PieA2 | BGC0000124 | Piericidin\_A1\_biosynthetic\_gene\_cluster | 30 | 34.9961028839 | 372 | 4.7e-102 |
| AAC68815.1 | FK506\_polyketide\_synthase | BGC0000353 | FK506\_biosynthetic\_gene\_cluster | 30 | 39.1660171473 | 372 | 4.7e-102 |
| CAL58684.1 | polyketide\_synthase | BGC0000149 | Spirangien\_biosynthetic\_gene\_cluster | 32 | 35.7755261107 | 372 | 4.7e-102 |
| ABK32259.1 | AmbE | BGC0000014 | Ambruticin\_biosynthetic\_gene\_cluster | 29 | 45.3234606391 | 372 | 4.7e-102 |
| CAJ88187.2 | putative\_type\_I\_modular\_polyketide\_synthase | BGC0000151 | Stambomycin\_biosynthetic\_gene\_cluster | 30 | 44.4660950896 | 371 | 6.2e-102 |
| ACB46487.1 | polyketide\_synthase | BGC0000082 | Kijanimicin\_biosynthetic\_gene\_cluster | 33 | 34.7622759158 | 371 | 6.2e-102 |
| ctg1\_orf253 |  | BGC0001200 | Lorneic\_acid\_A\_biosynthetic\_gene\_cluster | 29 | 40.841777085 | 371 | 6.2e-102 |
| AAS79461.1 | polyketide\_synthase\_subunit | BGC0000035 | Chalcomycin\_biosynthetic\_gene\_cluster | 28 | 42.3616523772 | 371 | 6.2e-102 |
| CAQ18830.1 | polyketide\_synthase | BGC0000954 | Ajudazol\_biosynthetic\_gene\_cluster | 29 | 48.2073265783 | 371 | 1.1e-101 |
| AAG13918.1 | megalomicin\_6-deoxyerythronolide\_B\_synthase\_2 | BGC0000092 | Megalomicin\_biosynthetic\_gene\_cluster | 31 | 35.5416991426 | 371 | 1.1e-101 |
| AHE80994.1 | PieA4 | BGC0001169 | Piericidin\_A1\_biosynthetic\_gene\_cluster | 29 | 43.7646141855 | 370 | 1.4e-101 |
| IF55\_RS32375 | beta-ketoacyl\_synthase | BGC0001348 | JBIR-100\_biosynthetic\_gene\_cluster | 28 | 44.9337490257 | 370 | 1.4e-101 |
| CAJ46689.1 | polyketide\_synthase | BGC0000969 | Chondramide\_biosynthetic\_gene\_cluster | 31 | 35.697583788 | 370 | 1.4e-101 |
| AAO65796.1 | monensin\_polyketide\_synthase\_loading\_module\_and\_module\_1 | BGC0000100 | Monensin\_biosynthetic\_gene\_cluster | 32 | 36.7108339829 | 370 | 1.8e-101 |
| AKD43769.1 | HerA2 | BGC0001349 | Heronamide\_biosynthetic\_gene\_cluster | 31 | 38.5035074045 | 370 | 1.8e-101 |
| AAP42855.1 | NanA1 | BGC0000105 | Nanchangmycin\_biosynthetic\_gene\_cluster | 31 | 38.5035074045 | 370 | 1.8e-101 |
| ACB46488.1 | polyketide\_synthase | BGC0000082 | Kijanimicin\_biosynthetic\_gene\_cluster | 32 | 34.6843335931 | 370 | 1.8e-101 |
| ABV83230.1 | CppC | BGC0000116 | Nystatin-like\_Pseudonocardia\_polyene\_biosynthetic\_gene\_cluster | 32 | 39.2829306313 | 369 | 2.4e-101 |
| AEK75504.1 | type\_1\_polyketide\_synthase | BGC0000001 | Abyssomicin\_biosynthetic\_gene\_cluster | 31 | 34.2946219797 | 369 | 2.4e-101 |
| CAE14173.1 |  | BGC0000383 | Luminmycin\_biosynthetic\_gene\_cluster | 30 | 41.7381137958 | 369 | 2.4e-101 |
| CAQ18838.1 | polyketide\_synthase | BGC0000954 | Ajudazol\_biosynthetic\_gene\_cluster | 33 | 32.8916601715 | 369 | 2.4e-101 |
| ABK32287.1 | JerA | BGC0000080 | Jerangolid\_biosynthetic\_gene\_cluster | 32 | 35.2299298519 | 369 | 2.4e-101 |
| AJO72737.1 | Type\_I\_modular\_polyketide\_synthase | BGC0001381 | Brasilinolide\_biosynthetic\_gene\_cluster | 32 | 35.8924395947 | 369 | 2.4e-101 |
| EJK79843.1 | amino\_acid\_adenylation\_enzyme/thioester\_reductase\_family\_protein | BGC0000436 | Syringolin\_A\_biosynthetic\_gene\_cluster | 30 | 41.8550272798 | 369 | 3.1e-101 |
| AEZ53952.1 | polyketide\_synthase | BGC0000144 | Salinomycin\_biosynthetic\_gene\_cluster | 32 | 35.6196414653 | 369 | 3.1e-101 |
| AAC01711.1 | RifB | BGC0000136 | Rifamycin\_biosynthetic\_gene\_cluster | 31 | 35.4247856586 | 369 | 3.1e-101 |
| WP\_042799407.1 | polyketide\_synthase | BGC0001283 | Arsenopolyketides\_biosynthetic\_gene\_cluster | 29 | 38.6983632112 | 369 | 4e-101 |
| AAZ94386.1 | modular\_polyketide\_synthase | BGC0000040 | Concanamycin\_A\_biosynthetic\_gene\_cluster | 29 | 44.4271239283 | 368 | 6.8e-101 |
| AHH99923.1 | PKS\_I | BGC0000002 | Aculeximycin\_biosynthetic\_gene\_cluster | 30 | 40.3741231489 | 368 | 6.8e-101 |
| AHD05614.1 | putative\_non-ribosomal\_peptide\_ligase/\_polyketide\_synthase\_hybrid | BGC0001033 | Paenilamicin\_biosynthetic\_gene\_cluster | 32 | 36.0872954014 | 368 | 8.9e-101 |
| AAT70102.1 | CurG | BGC0001165 | Curacin\_biosynthetic\_gene\_cluster | 30 | 35.697583788 | 368 | 8.9e-101 |
| CUW01176.1 | Type\_I\_polyketide\_synthase | BGC0001359\_c2 | PM100117\_/\_PM100118\_biosynthetic\_gene\_cluster | 31 | 35.9703819174 | 368 | 8.9e-101 |
| AEE88283.1 | CurG | BGC0000976 | Curacin\_biosynthetic\_gene\_cluster | 30 | 35.697583788 | 368 | 8.9e-101 |
| AHA12078.1 | polyketide\_synthase\_type\_1 | BGC0001172 | Chlorizidine\_A\_biosynthetic\_gene\_cluster | 31 | 36.9446609509 | 368 | 8.9e-101 |
| AAS79463.1 | polyketide\_synthase\_subunit | BGC0000035 | Chalcomycin\_biosynthetic\_gene\_cluster | 30 | 35.9703819174 | 367 | 1.2e-100 |
| BAH02269.1 | polyketide\_synthase | BGC0000126 | Pladienolide\_biosynthetic\_gene\_cluster | 30 | 36.8667186282 | 367 | 1.5e-100 |
| AFD30954.1 | CrmA | BGC0000966 | Caerulomycin\_A\_biosynthetic\_gene\_cluster | 30 | 36.0093530787 | 367 | 1.5e-100 |
| AAZ94390.1 | modular\_polyketide\_synthase | BGC0000040 | Concanamycin\_A\_biosynthetic\_gene\_cluster | 30 | 34.8791893998 | 367 | 1.5e-100 |
| ALA09355.1 | type\_I\_modular\_PKS | BGC0001303 | Lobosamide\_biosynthetic\_gene\_cluster | 32 | 35.580670304 | 367 | 1.5e-100 |
| ACO94471.1 | polyketide\_synthase\_type\_I | BGC0000029 | BE-14106\_biosynthetic\_gene\_cluster | 28 | 43.6087295401 | 367 | 1.5e-100 |
| AHB82063.1 | polyketide\_synthase | BGC0001231 | Microsclerodermins\_biosynthetic\_gene\_cluster | 36 | 25.6430241621 | 367 | 1.5e-100 |
| BAQ25482.1 | type\_I\_polyketide\_synthase | BGC0001288 | Maklamicin\_biosynthetic\_gene\_cluster | 36 | 26.773187841 | 367 | 1.5e-100 |
| BAJ16468.1 | polyketide\_synthase | BGC0000058 | FD-891\_biosynthetic\_gene\_cluster | 31 | 36.1652377241 | 366 | 2e-100 |
| CAJ46690.1 | polyketide\_synthase | BGC0000969 | Chondramide\_biosynthetic\_gene\_cluster | 29 | 44.4660950896 | 366 | 2e-100 |
| AKD43522.1 | Type\_I\_polyketide\_synthase | BGC0001409 | Dutomycin\_biosynthetic\_gene\_cluster | 30 | 38.4255650818 | 366 | 2e-100 |
| AIT55262.1 | GulE | BGC0000072 | Gulmirecin\_biosynthetic\_gene\_cluster | 31 | 36.6718628215 | 366 | 2e-100 |
| AAF71776.1 | NysC | BGC0000115 | Nystatin\_biosynthetic\_gene\_cluster | 31 | 39.2829306313 | 366 | 2.6e-100 |
| BAO66529.1 | type\_I\_polyketide\_synthase | BGC0000042 | Cremimycin\_biosynthetic\_gene\_cluster | 32 | 35.1909586906 | 366 | 2.6e-100 |
| AHH99926.1 | PKS\_I | BGC0000002 | Aculeximycin\_biosynthetic\_gene\_cluster | 32 | 34.4894777864 | 366 | 3.4e-100 |
| AHB82057.1 | polyketide\_synthase | BGC0001019 | Microsclerodermins\_biosynthetic\_gene\_cluster | 31 | 35.0350740452 | 366 | 3.4e-100 |
| ACO94488.1 | polyketide\_synthase\_type\_I | BGC0000097 | ML-449\_biosynthetic\_gene\_cluster | 31 | 35.2689010133 | 366 | 3.4e-100 |
| AAY28227.1 | HbmAIII | BGC0000074 | Herbimycin\_biosynthetic\_gene\_cluster | 31 | 37.1395167576 | 366 | 3.4e-100 |
| CAM00062.1 | EryAI\_Erythromycin\_polyketide\_synthase\_modules\_1\_and\_2 | BGC0000055 | Erythromycin\_biosynthetic\_gene\_cluster | 31 | 35.697583788 | 366 | 3.4e-100 |
| BAE93725.1 | type\_I\_polyketide\_synthase | BGC0000164 | Tetronomycin\_biosynthetic\_gene\_cluster | 31 | 35.4247856586 | 366 | 3.4e-100 |
| AGY62754.1 | EbeB | BGC0000051 | Ebelactone\_biosynthetic\_gene\_cluster | 29 | 44.3102104443 | 366 | 3.4e-100 |
| AFL48529.1 | laidlomycin\_polyketide\_synthase\_(module\_5\_and\_module\_6) | BGC0000084 | Laidlomycin\_biosynthetic\_gene\_cluster | 28 | 49.0257209665 | 365 | 4.4e-100 |
| AAK57190.1 | MxaF | BGC0001022 | Myxalamid\_biosynthetic\_gene\_cluster | 33 | 32.7747466875 | 365 | 4.4e-100 |
| BAQ21948.1 | putative\_type\_I\_polyketide\_synthase | BGC0001204 | Versipelostatin\_biosynthetic\_gene\_cluster | 32 | 35.4247856586 | 365 | 4.4e-100 |
| ABC84456.1 | NigAI | BGC0000114 | Nigericin\_biosynthetic\_gene\_cluster | 32 | 35.5416991426 | 365 | 4.4e-100 |
| ALA09358.1 | type\_I\_modular\_PKS | BGC0001303 | Lobosamide\_biosynthetic\_gene\_cluster | 33 | 35.0740452065 | 365 | 4.4e-100 |
| BAO66528.1 | type\_I\_polyketide\_synthase | BGC0000042 | Cremimycin\_biosynthetic\_gene\_cluster | 31 | 37.8020265004 | 365 | 5.8e-100 |
| CUW01173.1 | Type\_I\_polyketide\_synthase | BGC0001359 | PM100117\_/\_PM100118\_biosynthetic\_gene\_cluster | 29 | 41.6212003118 | 364 | 7.6e-100 |
| ABB05102.1 | LipPks1 | BGC0001003 | Lipomycin\_biosynthetic\_gene\_cluster | 31 | 35.5416991426 | 364 | 9.9e-100 |
| ABB52544.1 | putative\_type\_I\_polyketide\_synthase | BGC0000047 | Dihydrochalcomycin\_biosynthetic\_gene\_cluster | 31 | 34.9571317225 | 364 | 9.9e-100 |
| CQR60496.1 | Polyketide\_synthase,\_type\_I,\_modules:\_4,\_5\_and\_6 | BGC0001287 | Chaxamycin\_A\_/\_chaxamycin\_B\_/\_chaxamycin\_C\_/\_chaxamycin\_D | 30 | 40.841777085 | 364 | 1.3e-99 |
| AHA38199.1 | GphF | BGC0000069 | Gephyronic\_acid\_biosynthetic\_gene\_cluster | 27 | 51.5198752923 | 363 | 1.7e-99 |
| ACY06287.1 | type\_I\_polyketide\_synthase | BGC0001042 | Sanglifehrin\_A\_biosynthetic\_gene\_cluster | 32 | 36.2431800468 | 363 | 1.7e-99 |
| AHD05615.1 | putative\_non-ribosomal\_peptide\_ligase/\_polyketide\_synthase\_hybrid | BGC0001033 | Paenilamicin\_biosynthetic\_gene\_cluster | 31 | 35.1130163679 | 363 | 2.2e-99 |
| CAC20921.1 | PimS2\_protein | BGC0000125 | Pimaricin\_biosynthetic\_gene\_cluster | 31 | 35.3468433359 | 363 | 2.2e-99 |
| CAQ64691.1 | lasalocid\_modular\_polyketide\_synthase | BGC0000087 | Lasalocid\_biosynthetic\_gene\_cluster | 28 | 44.5440374123 | 363 | 2.2e-99 |
| AAF15892.2 | NosB | BGC0001028 | Nostopeptolide\_biosynthetic\_gene\_cluster | 30 | 35.6196414653 | 363 | 2.2e-99 |
| ABI91470.1 | beta-ketoacyl\_synthase | BGC0001094 | Enacyloxin\_biosynthetic\_gene\_cluster | 29 | 38.9321901793 | 363 | 2.9e-99 |
| CAL58682.1 | polyketide\_synthase | BGC0000149 | Spirangien\_biosynthetic\_gene\_cluster | 31 | 35.8924395947 | 363 | 2.9e-99 |
| EFL02193.1 | amino\_acid\_adenylation\_domain-containing\_protein | BGC0000996 | Frontalamides\_biosynthetic\_gene\_cluster | 32 | 35.3858144973 | 362 | 3.8e-99 |
| AAQ82561.1 | FscA | BGC0000034 | Candicidin\_biosynthetic\_gene\_cluster | 30 | 42.9851909587 | 362 | 3.8e-99 |
| CAI94713.1 | putative\_polyketide\_synthase | BGC0000141 | Rubradirin\_biosynthetic\_gene\_cluster | 30 | 39.9064692128 | 362 | 3.8e-99 |
| ACN69988.1 | polyketide\_synthase | BGC0000079 | Indanomycin\_biosynthetic\_gene\_cluster | 30 | 40.3741231489 | 362 | 4.9e-99 |
| AAB66508.1 | tylactone\_synthase\_module\_7 | BGC0000166 | Tylactone\_biosynthetic\_gene\_cluster | 31 | 35.931410756 | 362 | 4.9e-99 |
| ABB88533.1 | polyketide\_synthase\_type\_I | BGC0000049 | E-492\_/\_E-975\_biosynthetic\_gene\_cluster | 30 | 34.9961028839 | 362 | 4.9e-99 |
| CAE45669.1 | borrelidin\_polyketide\_synthase,\_type\_I | BGC0000031 | Borrelidin\_biosynthetic\_gene\_cluster | 28 | 51.1301636789 | 361 | 6.4e-99 |
| CAO98852.1 | polyketide\_synthase\_AufI | BGC0000023 | Aurafuron\_biosynthetic\_gene\_cluster | 32 | 32.5019485581 | 361 | 6.4e-99 |
| BAC76491.1 | lankamycin\_synthase\_LkmAIII | BGC0000085 | Lankamycin\_biosynthetic\_gene\_cluster | 32 | 31.9563522993 | 361 | 8.4e-99 |
| AAO06918.1 | GdmAIII | BGC0000066 | Geldanamycin\_biosynthetic\_gene\_cluster | 30 | 37.0226032736 | 361 | 8.4e-99 |
| BAO66543.1 | type\_I\_polyketide\_synthase | BGC0000042 | Cremimycin\_biosynthetic\_gene\_cluster | 32 | 32.1122369447 | 361 | 1.1e-98 |
| AAQ82565.1 | FscB | BGC0000034 | Candicidin\_biosynthetic\_gene\_cluster | 31 | 35.46375682 | 361 | 1.1e-98 |
| ANC94963.1 | AlmHIV | BGC0001396 | Aldgamycin\_biosynthetic\_gene\_cluster | 31 | 35.697583788 | 361 | 1.1e-98 |
| AAZ94387.1 | modular\_polyketide\_synthase | BGC0000040 | Concanamycin\_A\_biosynthetic\_gene\_cluster | 30 | 36.5159781761 | 360 | 1.4e-98 |
| BAF85837.1 | modular\_polyketide\_synthase | BGC0000109 | Nemadectin\_biosynthetic\_gene\_cluster | 29 | 44.6609508963 | 360 | 1.4e-98 |
| ABB86410.1 | GelC | BGC0000067 | Geldanamycin\_biosynthetic\_gene\_cluster | 30 | 37.0226032736 | 360 | 1.4e-98 |
| BAG85031.1 | putative\_polyketide\_synthase | BGC0000086 | Lasalocid\_biosynthetic\_gene\_cluster | 28 | 44.855806703 | 360 | 1.9e-98 |
| AEZ53949.1 | polyketide\_synthase | BGC0000144 | Salinomycin\_biosynthetic\_gene\_cluster | 29 | 42.0888542479 | 360 | 1.9e-98 |
| AAG23262.1 | polyketide\_synthase\_extender\_modules\_8-10 | BGC0000148 | Spinosad\_biosynthetic\_gene\_cluster | 31 | 35.8534684334 | 360 | 1.9e-98 |
| CAE45671.1 | borrelidin\_polyketide\_synthase,\_type\_I | BGC0000031 | Borrelidin\_biosynthetic\_gene\_cluster | 30 | 40.2572096648 | 360 | 1.9e-98 |
| ADC45515.1 | modular\_polyketide\_synthase | BGC0000093 | Meilingmycin\_biosynthetic\_gene\_cluster | 29 | 44.1543257989 | 359 | 2.4e-98 |
| AAC38075.1 | polyketide\_synthase\_type\_I | BGC0000127 | Pyoluteorin\_biosynthetic\_gene\_cluster | 31 | 35.1519875292 | 359 | 3.2e-98 |
| CCA29203.1 | non-ribosomal\_peptide\_synthetase/polyketide\_synthase | BGC0000955 | Althiomycin\_biosynthetic\_gene\_cluster | 31 | 35.6586126267 | 359 | 3.2e-98 |
| AJO72734.1 | Type\_I\_modular\_polyketide\_synthase | BGC0001381 | Brasilinolide\_biosynthetic\_gene\_cluster | 31 | 34.8012470772 | 359 | 3.2e-98 |
| ABW96540.1 | type\_I\_modular\_polyketide\_synthase | BGC0000159 | Tautomycin\_biosynthetic\_gene\_cluster | 30 | 41.192517537 | 359 | 4.2e-98 |
| ACO94468.1 | polyketide\_synthase\_type\_I | BGC0000029 | BE-14106\_biosynthetic\_gene\_cluster | 28 | 44.2712392829 | 359 | 4.2e-98 |
| ABV83221.1 | CppI | BGC0000116 | Nystatin-like\_Pseudonocardia\_polyene\_biosynthetic\_gene\_cluster | 32 | 35.7365549493 | 359 | 4.2e-98 |
| CQR60495.1 | Polyketide\_synthase,\_type\_I,\_module\_7 | BGC0001287 | Chaxamycin\_A\_/\_chaxamycin\_B\_/\_chaxamycin\_C\_/\_chaxamycin\_D | 31 | 34.9961028839 | 359 | 4.2e-98 |
| ADB23403.1 | polyketide\_synthase\_type\_I | BGC0001062 | TLN-05220\_biosynthetic\_gene\_cluster | 32 | 35.7755261107 | 359 | 4.2e-98 |
| ACN64831.1 | PokM1 | BGC0001061 | Polyketomycin\_biosynthetic\_gene\_cluster | 33 | 30.202650039 | 359 | 4.2e-98 |
| AAF71767.1 | NysJ | BGC0000115 | Nystatin\_biosynthetic\_gene\_cluster | 32 | 34.9571317225 | 359 | 4.2e-98 |
| AAP85335.1 | type\_I\_PKS | BGC0000233 | Hedamycin\_biosynthetic\_gene\_cluster | 29 | 44.1932969602 | 358 | 5.4e-98 |
| CAM00065.1 | EryAIII\_Erythromycin\_polyketide\_synthase\_modules\_5\_and\_6 | BGC0000055 | Erythromycin\_biosynthetic\_gene\_cluster | 30 | 34.9181605612 | 358 | 7.1e-98 |
| AEZ53945.1 | polyketide\_synthase | BGC0000144 | Salinomycin\_biosynthetic\_gene\_cluster | 30 | 39.127045986 | 358 | 7.1e-98 |
| ABB88519.1 | polyketide\_synthase\_type\_I | BGC0000050 | E-837\_biosynthetic\_gene\_cluster | 30 | 38.5814497272 | 358 | 7.1e-98 |
| AAK73513.1 | AmphB | BGC0000015 | Amphotericin\_biosynthetic\_gene\_cluster | 30 | 35.6586126267 | 358 | 7.1e-98 |
| ABV97154.1 | Beta-ketoacyl\_synthase | BGC0000137 | Rifamycin\_biosynthetic\_gene\_cluster | 30 | 39.7895557288 | 358 | 7.1e-98 |
| AFL48532.1 | laidlomycin\_polyketide\_synthase\_(module\_11\_and\_module\_12) | BGC0000084 | Laidlomycin\_biosynthetic\_gene\_cluster | 30 | 38.2307092751 | 358 | 9.3e-98 |
| ACF35447.1 | MbcAIII | BGC0000090 | Macbecin\_biosynthetic\_gene\_cluster | 30 | 39.2829306313 | 358 | 9.3e-98 |
| CAF05651.1 | TubF\_protein | BGC0001053 | Tubulysin\_biosynthetic\_gene\_cluster | 30 | 35.46375682 | 358 | 9.3e-98 |
| AFP87524.1 | type\_I\_polyketide\_synthase | BGC0001159 | Marinopyrrole\_biosynthetic\_gene\_cluster | 30 | 38.6204208885 | 358 | 9.3e-98 |
| ACF35446.1 | MbcAII | BGC0000090 | Macbecin\_biosynthetic\_gene\_cluster | 32 | 35.697583788 | 358 | 9.3e-98 |
| AAS79462.1 | polyketide\_synthase\_subunit | BGC0000035 | Chalcomycin\_biosynthetic\_gene\_cluster | 30 | 35.6196414653 | 358 | 9.3e-98 |
| CAF05649.1 | TubD\_protein | BGC0001053 | Tubulysin\_biosynthetic\_gene\_cluster | 32 | 35.3858144973 | 357 | 1.2e-97 |
| ADH04680.1 | hybrid\_polyketide\_synthase/non-ribosomal\_peptide\_synthetase | BGC0001344 | Tubulysin\_biosynthetic\_gene\_cluster | 32 | 36.6328916602 | 357 | 1.2e-97 |
| ADC79620.1 | BafAV | BGC0000028 | Bafilomycin\_biosynthetic\_gene\_cluster | 30 | 39.5557287607 | 357 | 1.2e-97 |
| ABC84460.1 | NigAV | BGC0000114 | Nigericin\_biosynthetic\_gene\_cluster | 30 | 35.3858144973 | 357 | 1.2e-97 |
| CBA11583.1 | polyketide\_synthase\_type\_I | BGC0001046 | Streptolydigin\_biosynthetic\_gene\_cluster | 29 | 39.5946999221 | 357 | 1.6e-97 |
| ABY21538.1 | AngAI | BGC0000018 | Angolamycin\_biosynthetic\_gene\_cluster | 31 | 35.7755261107 | 357 | 1.6e-97 |
| BAB69195.1 | modular\_polyketide\_synthase | BGC0000117 | Oligomycin\_biosynthetic\_gene\_cluster | 31 | 35.931410756 | 357 | 1.6e-97 |
| AAP42856.1 | NanA2 | BGC0000105 | Nanchangmycin\_biosynthetic\_gene\_cluster | 27 | 45.8690568979 | 356 | 2.1e-97 |
| CAO85896.1 | protein\_modular\_polyketide\_synthase\_NorA' | BGC0000110 | Neoaureothin\_biosynthetic\_gene\_cluster | 28 | 44.2322681216 | 356 | 2.1e-97 |
| ABG02264.1 | SalB | BGC0000143 | Salinomycin\_biosynthetic\_gene\_cluster | 31 | 35.3078721746 | 356 | 2.7e-97 |
| ADZ24995.1 | non-ribosomal\_peptide\_synthase/polyketide\_synthase | BGC0000380 | Leupyrrin\_biosynthetic\_gene\_cluster | 30 | 39.5167575994 | 356 | 2.7e-97 |
| AHD05619.1 | putative\_polyketide\_synthase\_subunit | BGC0001033 | Paenilamicin\_biosynthetic\_gene\_cluster | 30 | 35.3468433359 | 356 | 2.7e-97 |
| AAU93805.2 | polyketide\_synthase\_modules\_5\_and\_6 | BGC0000054 | Erythromycin\_biosynthetic\_gene\_cluster | 30 | 35.7365549493 | 356 | 3.5e-97 |
| AAP42873.1 | NanA11 | BGC0000105 | Nanchangmycin\_biosynthetic\_gene\_cluster | 31 | 35.3858144973 | 356 | 3.5e-97 |
| AEZ53951.1 | polyketide\_synthase | BGC0000144 | Salinomycin\_biosynthetic\_gene\_cluster | 31 | 34.9181605612 | 356 | 3.5e-97 |
| AGI99497.1 | type\_I\_polyketide\_synthase | BGC0001004 | Lobophorin\_biosynthetic\_gene\_cluster | 31 | 37.1395167576 | 355 | 4.6e-97 |
| AEU17898.1 | putative\_type\_I\_PKS | BGC0001072 | Simocyclinone\_biosynthetic\_gene\_cluster | 31 | 36.0483242401 | 355 | 4.6e-97 |
| AAM81584.2 | putative\_type\_I\_polyketide\_synthase | BGC0000047 | Dihydrochalcomycin\_biosynthetic\_gene\_cluster | 31 | 35.0740452065 | 355 | 4.6e-97 |
| BAE93731.1 | type\_I\_polyketide\_synthase | BGC0000164 | Tetronomycin\_biosynthetic\_gene\_cluster | 30 | 41.309431021 | 354 | 7.8e-97 |
| ACB46471.1 | polyketide\_synthase | BGC0000082 | Kijanimicin\_biosynthetic\_gene\_cluster | 30 | 39.6726422447 | 354 | 7.8e-97 |
| CAO91861.1 | putative\_PKS-NRPS\_protein | BGC0000968 | Chaetoglobosin\_biosynthetic\_gene\_cluster | 33 | 30.9820732658 | 354 | 7.8e-97 |
| ABV83223.1 | CppK | BGC0000116 | Nystatin-like\_Pseudonocardia\_polyene\_biosynthetic\_gene\_cluster | 32 | 34.8791893998 | 354 | 7.8e-97 |
| ABL74938.1 | TlmVIII | BGC0001048 | Tallysomycin\_biosynthetic\_gene\_cluster | 31 | 34.9181605612 | 354 | 7.8e-97 |
| ABY66019.1 | 6-methylsalicylic\_acid\_synthase | BGC0001008 | Maduropeptin\_biosynthetic\_gene\_cluster | 34 | 31.6445830086 | 354 | 7.8e-97 |
| CAQ64689.1 | lasalocid\_modular\_polyketide\_synthase | BGC0000087 | Lasalocid\_biosynthetic\_gene\_cluster | 31 | 34.9961028839 | 354 | 1e-96 |
| AGI61662.1 | modular\_PKS | BGC0000095\_c5 | Micromonolactam\_biosynthetic\_gene\_cluster | 32 | 35.5416991426 | 354 | 1e-96 |
| BAG85029.1 | putative\_polyketide\_synthase | BGC0000086 | Lasalocid\_biosynthetic\_gene\_cluster | 31 | 34.9961028839 | 354 | 1e-96 |
| XP\_011392701.1 | hypothetical\_protein | BGC0001281 | Ustilagic\_acid\_biosynthetic\_gene\_cluster | 27 | 50.9742790335 | 354 | 1e-96 |
| ABC84458.1 | NigAIII | BGC0000114 | Nigericin\_biosynthetic\_gene\_cluster | 29 | 42.4785658613 | 354 | 1.3e-96 |
| AAG02357.1 | polyketide\_synthase | BGC0000963 | Bleomycin\_biosynthetic\_gene\_cluster | 32 | 35.5416991426 | 354 | 1.3e-96 |
| AAF86392.1 | FkbC | BGC0000994 | FK520\_biosynthetic\_gene\_cluster | 30 | 40.4520654716 | 354 | 1.3e-96 |
| ABB88535.1 | polyketide\_synthase\_type\_I | BGC0000049\_c2 | E-492\_/\_E-975\_biosynthetic\_gene\_cluster | 31 | 32.3460639127 | 353 | 1.7e-96 |
| AJO72742.1 | Type\_I\_modular\_polyketide\_synthase | BGC0001381 | Brasilinolide\_biosynthetic\_gene\_cluster | 32 | 29.9298519096 | 353 | 2.3e-96 |
| ACR50773.1 | polyketide\_synthase | BGC0000163 | Tetronasin\_biosynthetic\_gene\_cluster | 30 | 38.6593920499 | 353 | 2.3e-96 |
| ABV83229.1 | CppB | BGC0000116 | Nystatin-like\_Pseudonocardia\_polyene\_biosynthetic\_gene\_cluster | 32 | 32.3460639127 | 352 | 3e-96 |
| AAS79459.1 | polyketide\_synthase\_subunit | BGC0000035 | Chalcomycin\_biosynthetic\_gene\_cluster | 28 | 44.5830085737 | 352 | 3e-96 |
| AHB82072.1 | non\_ribosomal\_peptide\_synthetase/polyketide\_synthase | BGC0001231 | Microsclerodermins\_biosynthetic\_gene\_cluster | 31 | 36.4380358535 | 352 | 3e-96 |
| ABI93779.1 | GdmPKS | BGC0000068 | Geldanamycin\_biosynthetic\_gene\_cluster | 31 | 35.580670304 | 352 | 3e-96 |
| ADA82585.1 | hybrid\_trans-AT\_polyketide\_synthase\_-\_nonribosomal\_peptide\_synthetase | BGC0001110 | Psymberin\_biosynthetic\_gene\_cluster | 36 | 24.7856586126 | 352 | 3e-96 |
| BAC57028.1 | protomycinolide\_IV\_synthase\_1 | BGC0000102 | Mycinamicin\_biosynthetic\_gene\_cluster | 29 | 37.1784879189 | 352 | 3.9e-96 |
| AEZ64505.1 | Herb | BGC0001065 | Herboxidiene\_biosynthetic\_gene\_cluster | 30 | 35.8534684334 | 352 | 3.9e-96 |
| AAR87760.2 | ZmaK | BGC0001059 | Zwittermycin\_A\_biosynthetic\_gene\_cluster | 31 | 35.1909586906 | 352 | 5.1e-96 |
| AAC46026.1 | polyketide\_synthase\_modules\_4\_and\_5 | BGC0000113 | Niddamycin\_biosynthetic\_gene\_cluster | 29 | 35.2299298519 | 352 | 5.1e-96 |
| AAB66507.1 | tylactone\_synthase\_module\_6 | BGC0000166 | Tylactone\_biosynthetic\_gene\_cluster | 32 | 35.931410756 | 352 | 5.1e-96 |
| CAQ52626.1 | type\_I\_polyketide\_synthase,\_loading\_module\_and\_modules\_1-3 | BGC0001066 | Kendomycin\_biosynthetic\_gene\_cluster | 29 | 43.5307872175 | 351 | 8.7e-96 |
| AKL78824.1 | GLPKS3 | BGC0001187 | Xenolozoyenone\_biosynthetic\_gene\_cluster | 31 | 34.099766173 | 351 | 8.7e-96 |
| BAQ21940.1 | putative\_Type\_I\_polyketide\_synthase | BGC0001204 | Versipelostatin\_biosynthetic\_gene\_cluster | 30 | 38.5424785659 | 351 | 8.7e-96 |
| ACZ65476.1 | type\_I\_modular\_polyketide\_synthase | BGC0000140 | RK-682\_biosynthetic\_gene\_cluster | 31 | 32.3070927514 | 351 | 8.7e-96 |
| AGI99482.1 | Type\_I\_polyketide\_synthase | BGC0001004 | Lobophorin\_biosynthetic\_gene\_cluster | 29 | 44.6609508963 | 351 | 1.1e-95 |
| CUW01172.1 | Type\_I\_polyketide\_synthase | BGC0001359 | PM100117\_/\_PM100118\_biosynthetic\_gene\_cluster | 29 | 35.0740452065 | 350 | 1.5e-95 |
| AAM54076.1 | polyketide\_synthase | BGC0000020 | Ansamitocin\_biosynthetic\_gene\_cluster | 31 | 35.5027279813 | 350 | 1.5e-95 |
| AHB82059.1 | non\_ribosomal\_peptide\_synthetase/polyketide\_synthase | BGC0001019 | Microsclerodermins\_biosynthetic\_gene\_cluster | 30 | 35.6586126267 | 350 | 1.5e-95 |
| AAQ90173.1 | polyketide\_synthase\_type\_I | BGC0000128 | Pyoluteorin\_biosynthetic\_gene\_cluster | 32 | 32.5798908807 | 350 | 1.5e-95 |
| ADC79616.1 | BafAI | BGC0000028 | Bafilomycin\_biosynthetic\_gene\_cluster | 30 | 34.9961028839 | 350 | 1.9e-95 |
| ABP55223.1 | beta-ketoacyl\_synthase | BGC0000142 | Salinilactam\_biosynthetic\_gene\_cluster | 32 | 35.0350740452 | 350 | 1.9e-95 |
| AAW03325.1 | CtaB | BGC0000982 | Cystothiazole\_A\_biosynthetic\_gene\_cluster | 29 | 37.0226032736 | 350 | 1.9e-95 |
| ABV83228.1 | CppA | BGC0000116 | Nystatin-like\_Pseudonocardia\_polyene\_biosynthetic\_gene\_cluster | 31 | 35.1909586906 | 349 | 2.5e-95 |
| CAQ52624.1 | type\_I\_polyketide\_synthase,\_modules\_7-8 | BGC0001066 | Kendomycin\_biosynthetic\_gene\_cluster | 30 | 39.24395947 | 349 | 4.3e-95 |
| CAI94682.1 | putative\_polyketide\_synthase | BGC0000141 | Rubradirin\_biosynthetic\_gene\_cluster | 31 | 35.697583788 | 348 | 5.6e-95 |
| AAM54075.1 | polyketide\_synthase | BGC0000020 | Ansamitocin\_biosynthetic\_gene\_cluster | 29 | 43.8815276695 | 348 | 5.6e-95 |
| AEC13070.1 | fosD | BGC0000060 | Fostriecin\_biosynthetic\_gene\_cluster | 31 | 36.2431800468 | 348 | 7.3e-95 |
| ABF87031.1 | non-ribosomal\_peptide\_synthetase/polyketide\_synthase | BGC0000393 | Myxoprincomide\_biosynthetic\_gene\_cluster | 30 | 36.2042088854 | 348 | 7.3e-95 |
| CAJ76298.1 | putative\_hybrid\_polyketide-non-ribosomal\_peptide\_synthetase | BGC0000972 | Colibactin\_biosynthetic\_gene\_cluster | 31 | 35.2689010133 | 348 | 7.3e-95 |
| CAE02606.1 | polyketide\_synthase\_type\_I | BGC0000024 | Aureothin\_biosynthetic\_gene\_cluster | 30 | 35.4247856586 | 347 | 9.6e-95 |
| AAO06916.1 | GdmAI | BGC0000066 | Geldanamycin\_biosynthetic\_gene\_cluster | 30 | 36.2042088854 | 347 | 9.6e-95 |
| AAF71775.1 | NysB | BGC0000115 | Nystatin\_biosynthetic\_gene\_cluster | 32 | 30.1636788776 | 347 | 9.6e-95 |
| ADX66470.1 | ScnS0 | BGC0000108 | Natamycin\_biosynthetic\_gene\_cluster | 31 | 35.6586126267 | 347 | 9.6e-95 |
| AHB82065.1 | polyketide\_synthase | BGC0001231 | Microsclerodermins\_biosynthetic\_gene\_cluster | 35 | 25.5261106781 | 347 | 9.6e-95 |
| ABJ97438.1 | MerB | BGC0001012 | Meridamycin\_biosynthetic\_gene\_cluster | 30 | 39.7116134061 | 347 | 9.6e-95 |
| ctg1\_orf21 |  | BGC0001013 | Meridamycin\_biosynthetic\_gene\_cluster | 30 | 39.4388152767 | 347 | 1.3e-94 |
| ABC87510.1 | polyketide\_synthase | BGC0001011 | Meridamycin\_biosynthetic\_gene\_cluster | 30 | 39.4388152767 | 347 | 1.3e-94 |
| AAC69331.1 | type\_I\_polyketide\_synthase\_PikAIII | BGC0000094 | Methymycin\_/\_pikromycin\_biosynthetic\_gene\_cluster | 31 | 35.5027279813 | 347 | 1.3e-94 |
| AAU93807.2 | polyketide\_synthase\_modules\_1\_and\_2 | BGC0000054 | Erythromycin\_biosynthetic\_gene\_cluster | 30 | 35.580670304 | 346 | 2.8e-94 |
| ACY13414.1 | amino\_acid\_adenylation\_domain\_protein | BGC0001367 | Haliamide\_biosynthetic\_gene\_cluster | 29 | 44.1932969602 | 346 | 3.6e-94 |
| ACY06289.1 | type\_I\_polyketide\_synthase | BGC0001042 | Sanglifehrin\_A\_biosynthetic\_gene\_cluster | 29 | 44.1543257989 | 346 | 3.6e-94 |
| ADC79619.1 | BafAIV | BGC0000028 | Bafilomycin\_biosynthetic\_gene\_cluster | 30 | 34.7622759158 | 346 | 3.6e-94 |
| CCT72377.1 | probable\_polyketide\_synthase | BGC0001305 | Fujikurins\_biosynthetic\_gene\_cluster | 32 | 30.9431021044 | 345 | 4.8e-94 |
| ACO94499.1 | polyketide\_synthase\_type\_I | BGC0000097 | ML-449\_biosynthetic\_gene\_cluster | 29 | 39.2049883087 | 345 | 4.8e-94 |
| BAC76493.1 | lankamycin\_synthase\_LkmAI | BGC0000085 | Lankamycin\_biosynthetic\_gene\_cluster | 31 | 35.46375682 | 345 | 6.2e-94 |
| BAV32159.1 | polyketide\_synthase | BGC0001373 | Sordarin\_biosynthetic\_gene\_cluster | 34 | 29.3452844895 | 345 | 6.2e-94 |
| CAD89773.1 | MelB\_protein | BGC0001010 | Melithiazol\_biosynthetic\_gene\_cluster | 30 | 37.9189399844 | 344 | 8.1e-94 |
| AAG13917.1 | megalomicin\_6-deoxyerythronolide\_B\_synthase\_1 | BGC0000092 | Megalomicin\_biosynthetic\_gene\_cluster | 30 | 35.3468433359 | 344 | 8.1e-94 |
| EHA28244.1 | hypothetical\_protein | BGC0001143 | Azanigerone\_biosynthetic\_gene\_cluster | 32 | 29.9298519096 | 344 | 1.1e-93 |
| CAQ52622.1 | type\_I\_polyketide\_synthase,\_modules\_4-5 | BGC0001066 | Kendomycin\_biosynthetic\_gene\_cluster | 31 | 35.0740452065 | 344 | 1.4e-93 |
| EHA19289.1 | hypothetical\_protein | BGC0001124 | Pyranonigrin\_E\_biosynthetic\_gene\_cluster | 30 | 34.7622759158 | 344 | 1.4e-93 |
| ACF35445.1 | MbcAI | BGC0000090 | Macbecin\_biosynthetic\_gene\_cluster | 30 | 35.1130163679 | 344 | 1.4e-93 |
| WP\_043476516.1 | beta-ketoacyl\_synthase | BGC0001232 | Leucanicidin\_biosynthetic\_gene\_cluster | 32 | 31.8394388153 | 343 | 1.8e-93 |
| ctg1\_orf27 |  | BGC0000096 | Midecamycin\_biosynthetic\_gene\_cluster | 30 | 34.4115354638 | 343 | 2.4e-93 |
| IF55\_RS36525 | polyketide\_synthase | BGC0001348 | JBIR-100\_biosynthetic\_gene\_cluster | 31 | 36.3990646921 | 343 | 2.4e-93 |
| BAB69198.1 | modular\_polyketide\_synthase | BGC0000117 | Oligomycin\_biosynthetic\_gene\_cluster | 30 | 35.7365549493 | 342 | 4e-93 |
| CAQ52623.1 | type\_I\_polyketide\_synthase,\_module\_6 | BGC0001066 | Kendomycin\_biosynthetic\_gene\_cluster | 30 | 35.0350740452 | 342 | 5.3e-93 |
| AAF26920.1 | polyketide\_synthase | BGC0000988 | Epothilone\_biosynthetic\_gene\_cluster | 29 | 35.0740452065 | 342 | 5.3e-93 |
| CAO98848.1 | polyketide\_synthase\_AufE | BGC0000023 | Aurafuron\_biosynthetic\_gene\_cluster | 33 | 29.6570537802 | 341 | 6.9e-93 |
| AAK83194.1 | polyketide\_synthase | BGC0000026 | Avilamycin\_A\_biosynthetic\_gene\_cluster | 30 | 35.931410756 | 340 | 1.5e-92 |
| BAC57029.1 | protomycinolide\_IV\_synthase\_2 | BGC0000102 | Mycinamicin\_biosynthetic\_gene\_cluster | 29 | 38.5424785659 | 340 | 1.5e-92 |
| AAZ94388.1 | nodular\_polyketide\_synthase | BGC0000040 | Concanamycin\_A\_biosynthetic\_gene\_cluster | 29 | 34.9961028839 | 340 | 1.5e-92 |
| AEH42473.1 | polyketide\_synthase | BGC0000032 | Calcimycin\_biosynthetic\_gene\_cluster | 29 | 36.4380358535 | 340 | 1.5e-92 |
| AAV66110.2 | fusaridione\_A\_synthetase | BGC0000992 | Fusaridione\_A\_biosynthetic\_gene\_cluster | 31 | 33.9438815277 | 340 | 2e-92 |
| BAB69193.1 |  | BGC0000117 | Oligomycin\_biosynthetic\_gene\_cluster | 30 | 37.6071706937 | 340 | 2e-92 |
| CAL80821.1 | sylD-like\_NRPS/PKS | BGC0000997 | Glidobactin\_biosynthetic\_gene\_cluster | 30 | 35.3858144973 | 340 | 2e-92 |
| CBF87072.1 | polyketide\_synthase,\_putative\_(Eurofung) | BGC0001290 | Emericellamide\_A\_/\_emericellamide\_B\_biosynthetic\_gene\_cluster | 32 | 31.9563522993 | 339 | 3.4e-92 |
| AAZ77698.1 | ChlA5 | BGC0000036 | Chlorothricin\_biosynthetic\_gene\_cluster | 29 | 40.7248636009 | 338 | 5.8e-92 |
| BAQ25507.1 | type\_I\_polyketide\_synthase | BGC0001288 | Maklamicin\_biosynthetic\_gene\_cluster | 32 | 35.1519875292 | 338 | 5.8e-92 |
| AAF62882.1 | EpoC | BGC0000991 | Epothilone\_biosynthetic\_gene\_cluster | 29 | 35.0740452065 | 338 | 5.8e-92 |
| AAM70355.1 | CalO5 | BGC0000033 | Calicheamicin\_biosynthetic\_gene\_cluster | 32 | 31.0989867498 | 338 | 5.8e-92 |
| AAD03048.1 | type\_I\_polyketide\_synthase | BGC0000041 | Coronafacic\_acid\_biosynthetic\_gene\_cluster | 30 | 32.1901792673 | 338 | 5.8e-92 |
| CAE45670.1 | borrelidin\_polyketide\_synthase,\_type\_I | BGC0000031 | Borrelidin\_biosynthetic\_gene\_cluster | 30 | 34.7622759158 | 337 | 9.9e-92 |
| AEH42474.1 | polyketide\_synthase | BGC0000032 | Calcimycin\_biosynthetic\_gene\_cluster | 29 | 36.7108339829 | 337 | 1.7e-91 |
| AHB38498.1 | EpxE | BGC0000346 | Epoxomicin\_biosynthetic\_gene\_cluster | 31 | 34.8012470772 | 337 | 1.7e-91 |
| AAC69332.1 | type\_I\_polyketide\_synthase\_PikAIV | BGC0000094 | Methymycin\_/\_pikromycin\_biosynthetic\_gene\_cluster | 30 | 35.0350740452 | 336 | 2.2e-91 |
| ABW96542.1 | type\_I\_modular\_polyketide\_synthase | BGC0000159 | Tautomycin\_biosynthetic\_gene\_cluster | 35 | 26.2275915822 | 336 | 2.9e-91 |
| BAK26562.1 | PKS-NRPS\_hybrid | BGC0000977 | Cyclopiazonic\_acid\_biosynthetic\_gene\_cluster | 31 | 33.3203429462 | 336 | 3.8e-91 |
| AAU93806.2 | polyketide\_synthase\_modules\_3\_and\_4 | BGC0000054 | Erythromycin\_biosynthetic\_gene\_cluster | 29 | 39.2829306313 | 336 | 3.8e-91 |
| BAK64649.1 | polyketide\_synthase | BGC0000135 | Reveromycin\_biosynthetic\_gene\_cluster | 30 | 36.0093530787 | 335 | 4.9e-91 |
| ADB12490.1 | EpoC | BGC0000990 | Epothilone\_biosynthetic\_gene\_cluster | 29 | 35.0740452065 | 335 | 4.9e-91 |
| CBW54671.1 | polyketide\_synthase/non\_ribosomal\_peptide\_synthetase | BGC0000971 | Cinnabaramide\_biosynthetic\_gene\_cluster | 32 | 30.553390491 | 335 | 4.9e-91 |
| AJO72735.1 | Type\_I\_modular\_polyketide\_synthase | BGC0001381 | Brasilinolide\_biosynthetic\_gene\_cluster | 29 | 36.0483242401 | 335 | 6.4e-91 |
| AHA38202.1 | GphI | BGC0000069 | Gephyronic\_acid\_biosynthetic\_gene\_cluster | 32 | 31.8394388153 | 334 | 8.4e-91 |
| ADU86003.1 | putative\_modular\_polyketide\_synthase | BGC0000165 | Tiacumicin\_B\_biosynthetic\_gene\_cluster | 30 | 35.697583788 | 334 | 1.1e-90 |
| ctg1\_orf255 |  | BGC0001200 | Lorneic\_acid\_A\_biosynthetic\_gene\_cluster | 27 | 41.5822291504 | 334 | 1.1e-90 |
| ANC94966.1 | AlmHI | BGC0001396 | Aldgamycin\_biosynthetic\_gene\_cluster | 30 | 35.5027279813 | 334 | 1.1e-90 |
| BI06\_RS39060 | polyketide\_synthase | BGC0001232 | Leucanicidin\_biosynthetic\_gene\_cluster | 30 | 36.2431800468 | 334 | 1.4e-90 |
| ADU85988.1 | putative\_iterative\_type\_I\_polyketide\_synthase | BGC0000165 | Tiacumicin\_B\_biosynthetic\_gene\_cluster | 31 | 36.7887763055 | 334 | 1.4e-90 |
| ADF88265.1 | mixed\_nonribosomal\_peptide\_synthetase/\_polyketide\_synthase | BGC0000980 | Cylindrospermopsin\_biosynthetic\_gene\_cluster | 32 | 28.4099766173 | 333 | 1.9e-90 |
| AAB66506.1 | tylactone\_synthase\_modules\_4\_&\_5 | BGC0000166 | Tylactone\_biosynthetic\_gene\_cluster | 31 | 35.697583788 | 333 | 1.9e-90 |
| ADF88262.1 | mixed\_nonribosomal\_peptide\_synthetase/\_polyketide\_synthase | BGC0000979 | Cylindrospermopsin\_biosynthetic\_gene\_cluster | 32 | 28.4099766173 | 333 | 1.9e-90 |
| AHB82054.1 | polyketide\_synthase | BGC0001019 | Microsclerodermins\_biosynthetic\_gene\_cluster | 34 | 25.3702260327 | 333 | 1.9e-90 |
| AEZ53953.1 | polyketide\_synthase | BGC0000144 | Salinomycin\_biosynthetic\_gene\_cluster | 31 | 35.5027279813 | 333 | 2.4e-90 |
| CDG12864.1 | non-ribosomal\_peptide\_synthetase | BGC0001415 | Althiomycin\_biosynthetic\_gene\_cluster | 29 | 35.2299298519 | 333 | 2.4e-90 |
| EAQ86385.1 | hypothetical\_protein | BGC0001405 | Chaetoviridin\_/\_chaetomugilin\_biosynthetic\_gene\_cluster | 31 | 30.1247077163 | 332 | 3.2e-90 |
| ABA02240.1 | polyketide\_synthase | BGC0000098 | Monacolin\_K\_biosynthetic\_gene\_cluster | 32 | 29.9688230709 | 331 | 7.1e-90 |
| CAD70195.1 | non-ribosomal\_peptide\_synthetase | BGC0001047 | Syringolin\_A\_biosynthetic\_gene\_cluster | 30 | 41.6212003118 | 331 | 9.3e-90 |
| AAR16521.1 | RimA | BGC0000138 | Rimocidin\_biosynthetic\_gene\_cluster | 31 | 35.3468433359 | 331 | 9.3e-90 |
| AKA54627.1 | PKS | BGC0001216 | Splenocin\_biosynthetic\_gene\_cluster | 30 | 35.5027279813 | 329 | 3.5e-89 |
| CAQ18832.1 | polyketide\_synthase | BGC0000954 | Ajudazol\_biosynthetic\_gene\_cluster | 26 | 48.5580670304 | 329 | 4.6e-89 |
| BAJ14522.1 | polyketide\_synthase | BGC0001254 | ACT-Toxin\_II\_biosynthetic\_gene\_cluster | 32 | 29.4621979735 | 329 | 4.6e-89 |
| ABX37384.1 | Beta-ketoacyl\_synthase | BGC0000984 | Delftibactin\_biosynthetic\_gene\_cluster | 29 | 38.2307092751 | 329 | 4.6e-89 |
| EAU38971.1 | hypothetical\_protein | BGC0001122 | Isoflavipucine\_biosynthetic\_gene\_cluster | 29 | 35.580670304 | 328 | 6e-89 |
| AAB66504.1 | tylactone\_synthase\_starter\_module\_and\_modules\_1\_&\_2 | BGC0000166 | Tylactone\_biosynthetic\_gene\_cluster | 30 | 35.7365549493 | 328 | 7.9e-89 |
| ACB46194.1 | polyketide\_synthase | BGC0000989 | Epothilone\_biosynthetic\_gene\_cluster | 29 | 35.0740452065 | 327 | 1.7e-88 |
| ACZ57548.1 | polyketide\_synthase | BGC0000046 | Depudecin\_biosynthetic\_gene\_cluster | 28 | 47.2330475448 | 326 | 2.3e-88 |
| CBJ89764.1 | Polyketide\_synthase\_involved\_in\_xenocoumacin\_synthesis | BGC0001054 | Xenocoumacin\_biosynthetic\_gene\_cluster | 30 | 35.1130163679 | 325 | 5.1e-88 |
| AAT28740.1 | FUSS | BGC0000064 | Fusarin\_biosynthetic\_gene\_cluster | 31 | 30.0857365549 | 325 | 5.1e-88 |
| EWG54266.1 | hypothetical\_protein | BGC0001190 | Fusaric\_acid\_biosynthetic\_gene\_cluster | 32 | 29.8519095869 | 325 | 5.1e-88 |
| ABY83164.1 | Azi26 | BGC0000960 | Azinomycin\_B\_biosynthetic\_gene\_cluster | 29 | 40.3741231489 | 325 | 6.6e-88 |
| ALJ49921.1 | TtmH | BGC0001236 | Thiotetroamide\_biosynthetic\_gene\_cluster | 27 | 38.6593920499 | 324 | 8.7e-88 |
| ABP55222.1 | beta-ketoacyl\_synthase | BGC0000142 | Salinilactam\_biosynthetic\_gene\_cluster | 31 | 34.9181605612 | 324 | 1.1e-87 |
| WP\_010639241.1 | beta-ketoacyl\_synthase | BGC0000958 | Antimycin\_biosynthetic\_gene\_cluster | 29 | 36.1262665627 | 324 | 1.1e-87 |
| CCC55921.1 | non-ribosomal\_peptide\_synthetase/polyketide\_synthase\_hybrid\_protein | BGC0000973 | Collismycin\_A\_biosynthetic\_gene\_cluster | 29 | 36.4380358535 | 324 | 1.1e-87 |
| AFP73394.1 | FusA | BGC0001268 | Fusarin\_biosynthetic\_gene\_cluster | 31 | 30.2416212003 | 324 | 1.5e-87 |
| CBD77746.1 | non-ribosomal\_peptide\_synthetase/polyketide\_synthase | BGC0000974 | Crocacin\_biosynthetic\_gene\_cluster | 26 | 49.3374902572 | 323 | 1.9e-87 |
| ctg1\_orf0002 |  | BGC0001068 | Pyripyropene\_A\_biosynthetic\_gene\_cluster | 30 | 32.5019485581 | 323 | 2.5e-87 |
| AGO86662.1 | equisetin\_synthetase | BGC0001255 | Equisetin\_biosynthetic\_gene\_cluster | 31 | 29.9688230709 | 322 | 3.3e-87 |
| ACS68554.1 | hybrid\_PKS-NRPS\_protein | BGC0001026 | NG-391\_biosynthetic\_gene\_cluster | 32 | 29.5011691348 | 322 | 3.3e-87 |
| ALD83687.1 | tAT\_polyketide\_synthase | BGC0001300 | Anthracimycin\_biosynthetic\_gene\_cluster | 37 | 20.1480904131 | 322 | 5.6e-87 |
| AAN85523.1 | polyketide\_synthase | BGC0001101 | Leinamycin\_biosynthetic\_gene\_cluster | 38 | 20.5378020265 | 322 | 5.6e-87 |
| EAL85113.2 | hybrid\_PKS-NRPS\_enzyme,\_putative | BGC0001037 | Pseurotin\_A\_biosynthetic\_gene\_cluster | 31 | 30.5144193297 | 321 | 7.4e-87 |
| CUI25744.1 | Polyketide\_synthase/Nonribosomal\_peptide\_synthase | BGC0001354 | U-68204\_biosynthetic\_gene\_cluster | 28 | 35.580670304 | 321 | 9.6e-87 |
| CAL69597.1 | nonribosomal\_peptide\_synthase | BGC0001049 | Tenellin\_biosynthetic\_gene\_cluster | 31 | 30.7872174591 | 321 | 9.6e-87 |
| AFV52200.1 | polyketide\_synthase\_module | BGC0000081 | Kedarcidin\_biosynthetic\_gene\_cluster | 29 | 36.5939204988 | 321 | 1.3e-86 |
| CAQ34929.1 | putative\_polyketide\_synthase | BGC0000986 | Dkxanthene\_biosynthetic\_gene\_cluster | 29 | 36.5939204988 | 321 | 1.3e-86 |
| CAJ76291.1 | putative\_polyketide\_synthase | BGC0000972 | Colibactin\_biosynthetic\_gene\_cluster | 29 | 37.412314887 | 320 | 1.6e-86 |
| AHA38200.1 | GphG | BGC0000069 | Gephyronic\_acid\_biosynthetic\_gene\_cluster | 32 | 32.6188620421 | 320 | 1.6e-86 |
| ACR50795.1 | putative\_polyketide\_synthase | BGC0000163 | Tetronasin\_biosynthetic\_gene\_cluster | 34 | 25.3702260327 | 320 | 2.1e-86 |
| AIJ04684.1 | polyketide\_synthase | BGC0001383 | Macrolactin\_biosynthetic\_gene\_cluster | 36 | 20.1091192518 | 319 | 3.6e-86 |
| ABP53498.1 | beta-ketoacyl\_synthase | BGC0001041 | Salinosporamide\_biosynthetic\_gene\_cluster | 31 | 30.202650039 | 319 | 4.8e-86 |
| ADN68477.1 | SorB | BGC0000184 | Sorangicin\_biosynthetic\_gene\_cluster | 37 | 22.7591582229 | 318 | 6.2e-86 |
| BAC20566.1 | polyketide\_synthase | BGC0000039 | Compactin\_biosynthetic\_gene\_cluster | 31 | 29.3452844895 | 317 | 1.1e-85 |
| ABP73645.1 | SalA | BGC0000145 | Salinosporamide\_A\_biosynthetic\_gene\_cluster | 31 | 30.202650039 | 317 | 1.4e-85 |
| CAQ43079.1 | polyketide\_synthase | BGC0000970 | Chondrochloren\_biosynthetic\_gene\_cluster | 36 | 22.1745908028 | 316 | 2.4e-85 |
| EHA22196.1 | polyketide\_synthase | BGC0000170 | Yanuthone\_D\_biosynthetic\_gene\_cluster | 31 | 29.9298519096 | 316 | 2.4e-85 |
| CBF80487.1 | hybrid\_PKS-NRPS\_(Eurofung) | BGC0000959 | Aspyridone\_biosynthetic\_gene\_cluster | 31 | 30.6703039751 | 316 | 3.1e-85 |
| ABO15888.1 | polyketide\_synthase | BGC0000132 | Pyrrolomycin\_biosynthetic\_gene\_cluster | 32 | 31.7225253313 | 316 | 3.1e-85 |
| EAL89230.2 | LovB-like\_polyketide\_synthase,\_putative | BGC0000129 | Pyripyropene\_biosynthetic\_gene\_cluster | 29 | 32.4629773967 | 316 | 4e-85 |
| AFN27480.1 | pks\_BonA | BGC0000173 | Bongkrekic\_acid\_biosynthetic\_gene\_cluster | 35 | 21.3172252533 | 316 | 4e-85 |
| CCC21123.1 | type-I\_polyketide\_synthases | BGC0000171 | 9-methylstreptimidone\_biosynthetic\_gene\_cluster | 35 | 20.5378020265 | 315 | 5.3e-85 |
| CBK62733.1 |  | BGC0001115 | Thiomarinol\_biosynthetic\_gene\_cluster | 33 | 22.1356196415 | 314 | 9e-85 |
| CBJ89766.1 | Polyketide\_synthase\_involved\_in\_xenocoumacin\_synthesis | BGC0001054 | Xenocoumacin\_biosynthetic\_gene\_cluster | 28 | 36.5549493375 | 314 | 9e-85 |
| AIA58899.1 | HRPKS | BGC0001141 | Brefeldin\_biosynthetic\_gene\_cluster | 30 | 35.0740452065 | 314 | 9e-85 |
| BAQ25466.1 | polyketide\_synthase | BGC0001264 | Betaenone\_A\_/\_betaenone\_B\_/\_betaenone\_C\_biosynthetic\_gene\_cluster | 32 | 29.9298519096 | 314 | 9e-85 |
| CAG23977.1 | polyketide\_synthase\_type\_I | BGC0000176 | Difficidin\_biosynthetic\_gene\_cluster | 35 | 20.6547155105 | 314 | 1.5e-84 |
| CAA73127.1 | HMWP1\_protein | BGC0000467 | Yersiniabactin\_biosynthetic\_gene\_cluster | 27 | 36.8667186282 | 314 | 1.5e-84 |
| CUI25727.1 | Polyketide\_synthase/Nonribosomal\_peptide\_synthase | BGC0001355 | U-68204\_biosynthetic\_gene\_cluster | 28 | 38.9711613406 | 312 | 3.4e-84 |
| ADN68479.1 | SorD | BGC0000184 | Sorangicin\_biosynthetic\_gene\_cluster | 36 | 20.5767731878 | 312 | 5.8e-84 |
| CAJ87591.1 | putative\_peptide/polyketide\_synthetase | BGC0001055 | Yersiniabactin\_biosynthetic\_gene\_cluster | 27 | 37.0226032736 | 311 | 7.6e-84 |
| ALD83704.1 | tAT\_polyketide\_synthase | BGC0001299 | Chlorotonil\_biosynthetic\_gene\_cluster | 35 | 22.9150428683 | 311 | 7.6e-84 |
| AAX35547.1 | polyketide\_syntase\_2 | BGC0001275 | 6-methylsalicyclic\_acid\_biosynthetic\_gene\_cluster | 29 | 34.2556508184 | 311 | 9.9e-84 |
| AAQ84146.1 | Plm6 | BGC0000123 | Phoslactomycin\_B\_biosynthetic\_gene\_cluster | 29 | 35.1130163679 | 311 | 9.9e-84 |
| CAN93348.1 | polyketide\_synthase | BGC0000179 | Etnangien\_biosynthetic\_gene\_cluster | 34 | 21.0444271239 | 310 | 2.2e-83 |
| CAQ34917.1 | polyketide\_synthase | BGC0000986 | Dkxanthene\_biosynthetic\_gene\_cluster | 33 | 26.5003897116 | 309 | 3.8e-83 |
| AFX60311.1 | polyketide\_synthase | BGC0001031 | Oocydin\_A\_biosynthetic\_gene\_cluster | 36 | 22.0187061574 | 309 | 4.9e-83 |
| ACR50796.1 | putative\_polyketide\_synthase | BGC0000163 | Tetronasin\_biosynthetic\_gene\_cluster | 37 | 20.6157443492 | 309 | 4.9e-83 |
| KGO40478.1 | Acyl\_transferase/acyl\_hydrolase/lysophospholipase | BGC0001205 | Communesin\_biosynthetic\_gene\_cluster | 29 | 37.5681995323 | 308 | 6.4e-83 |
| EYT83439.1 | beta-ketoacyl\_synthase | BGC0001213 | Nataxazole\_biosynthetic\_gene\_cluster | 31 | 32.9306313328 | 307 | 1.1e-82 |
| CBF79143.1 | polyketide\_synthase,\_putative\_(JCVI) | BGC0000013 | Alternariol\_biosynthetic\_gene\_cluster | 30 | 35.5416991426 | 307 | 1.4e-82 |
| AGS06823.1 | type\_I\_polyketide\_synthase,\_PedI-like\_protein | BGC0001092 | Diaphorin\_biosynthetic\_gene\_cluster | 35 | 20.8106001559 | 306 | 3.2e-82 |
| AGN11881.1 | TstDEF | BGC0001114 | Thailanstatin\_biosynthetic\_gene\_cluster | 36 | 20.0701480904 | 305 | 5.5e-82 |
| CBJ89760.1 | Polyketide\_synthase\_involved\_in\_xenocoumacin\_synthesis | BGC0001054 | Xenocoumacin\_biosynthetic\_gene\_cluster | 29 | 32.2291504287 | 305 | 5.5e-82 |
| AMH40423.1 | PKS | BGC0001350 | Phormidolide\_biosynthetic\_gene\_cluster | 35 | 21.628994544 | 305 | 5.5e-82 |
| BAC20564.1 | polyketide\_synthase | BGC0000039 | Compactin\_biosynthetic\_gene\_cluster | 30 | 30.2416212003 | 304 | 9.3e-82 |
| AHV78252.1 | ResS1 | BGC0001246 | Trans-resorcylide\_biosynthetic\_gene\_cluster | 31 | 29.7739672642 | 304 | 9.3e-82 |
| BAD97694.1 | Aft9-1 | BGC0000003 | AF-toxin\_biosynthetic\_gene\_cluster | 32 | 26.0327357755 | 304 | 1.6e-81 |
| AAM77986.1 | iterative\_type\_I\_polyketide\_synthase | BGC0000112 | Neocarzinostatin\_biosynthetic\_gene\_cluster | 31 | 32.5019485581 | 302 | 6e-81 |
| CBW45741.1 | hybrid\_PKS/NRPS\_(PKSI) | BGC0000952\_c3 | Pristinamycin\_biosynthetic\_gene\_cluster | 36 | 20.0701480904 | 301 | 7.9e-81 |
| ACM79805.1 | ZmaA | BGC0001059 | Zwittermycin\_A\_biosynthetic\_gene\_cluster | 29 | 35.6196414653 | 301 | 7.9e-81 |
| CBW45749.1 | hybrid\_PKS/NRPS\_(PKSI) | BGC0000952\_c3 | Pristinamycin\_biosynthetic\_gene\_cluster | 36 | 21.0444271239 | 301 | 1e-80 |
| CBK62731.1 |  | BGC0001115 | Thiomarinol\_biosynthetic\_gene\_cluster | 34 | 22.2525331255 | 300 | 1.3e-80 |
| AFX60313.1 | polyketide\_synthase | BGC0001031 | Oocydin\_A\_biosynthetic\_gene\_cluster | 36 | 20.2260327358 | 300 | 2.3e-80 |
| BAP05593.1 | CalE | BGC0000967 | Calyculin\_biosynthetic\_gene\_cluster | 36 | 20.5378020265 | 299 | 3.9e-80 |
| AFX60336.1 | polyketide\_synthase | BGC0001032 | Oocydin\_A\_biosynthetic\_gene\_cluster | 36 | 20.4598597038 | 299 | 3.9e-80 |
| ctg1\_orf6 |  | BGC0001109 | Pederin\_biosynthetic\_gene\_cluster | 33 | 22.2915042868 | 299 | 5.1e-80 |
| AAS47564.1 | mixed\_type\_I\_polyketide\_synthase/nonribosomal\_peptide\_synthetase | BGC0001108 | Pederin\_biosynthetic\_gene\_cluster | 33 | 22.2915042868 | 299 | 5.1e-80 |
| EAA65604.1 | hypothetical\_protein | BGC0000022 | Asperfuranone\_biosynthetic\_gene\_cluster | 29 | 30.1636788776 | 298 | 6.7e-80 |
| AFB35635.1 | EsmB4 | BGC0000935 | Esmeraldin\_biosynthetic\_gene\_cluster | 32 | 25.9158222915 | 297 | 1.1e-79 |
| ABF89568.1 | polyketide\_synthase | BGC0001025 | Myxovirescin\_biosynthetic\_gene\_cluster | 35 | 21.161340608 | 296 | 2.5e-79 |
| ADD82940.1 | Bat2 | BGC0001099 | Kalimantacin\_/\_batumin\_biosynthetic\_gene\_cluster | 34 | 21.8238503507 | 294 | 9.6e-79 |
| AEP40932.1 | polyketide\_synthase\_type\_I | BGC0000021 | Apoptolidin\_biosynthetic\_gene\_cluster | 28 | 37.6851130164 | 292 | 3.7e-78 |
| AFK83820.1 | PtzD | BGC0001107\_c2 | Patellazole\_biosynthetic\_gene\_cluster | 33 | 23.226812159 | 292 | 6.2e-78 |
| AEC04364.1 | polyketide\_synthase | BGC0000178 | Elansolid\_biosynthetic\_gene\_cluster | 35 | 20.3039750585 | 289 | 4e-77 |
| AGS06824.1 | mixed\_type\_I\_polyketide\_synthase/non-ribosomal\_peptide\_synthetase,\_PedH-like\_protein | BGC0001092 | Diaphorin\_biosynthetic\_gene\_cluster | 34 | 20.3429462198 | 287 | 1.2e-76 |
| ADN43685.1 | DmbS | BGC0001136 | Desmethylbassianin\_biosynthetic\_gene\_cluster | 35 | 20.3429462198 | 279 | 5.5e-74 |
| AEF33079.1 | polyketide\_synthase | BGC0001039 | Pyridomycin\_biosynthetic\_gene\_cluster | 29 | 35.580670304 | 278 | 7.1e-74 |
| AAV97877.1 | OnnI | BGC0001105 | Onnamide\_biosynthetic\_gene\_cluster | 32 | 21.9797349961 | 276 | 3.5e-73 |
| ACN69992.1 | polyketide\_synthase | BGC0000079 | Indanomycin\_biosynthetic\_gene\_cluster | 31 | 25.8768511302 | 275 | 7.9e-73 |
| AKA59437.1 | polyketide\_synthase | BGC0001202 | Landepoxcin\_biosynthetic\_gene\_cluster | 31 | 27.6695245518 | 275 | 7.9e-73 |
| CAJ57410.1 | polyketide\_synthase\_type\_I | BGC0000176 | Difficidin\_biosynthetic\_gene\_cluster | 34 | 21.2392829306 | 274 | 1e-72 |
| AKA59448.1 | polyketide\_synthase | BGC0001203 | Clarexpoxcin\_biosynthetic\_gene\_cluster | 29 | 36.0483242401 | 273 | 2.3e-72 |
| ACG60781.1 | PKS(KS/AT/MT/KR/ACP) | BGC0001058 | Zorbamycin\_biosynthetic\_gene\_cluster | 33 | 25.6040530008 | 273 | 2.3e-72 |
| AHD05620.1 | putative\_polyketide\_synthase\_subunit | BGC0001033 | Paenilamicin\_biosynthetic\_gene\_cluster | 31 | 25.2533125487 | 269 | 3.3e-71 |
| ABS90478.1 | PKS | BGC0001106 | Oxazolomycin\_biosynthetic\_gene\_cluster | 33 | 22.3304754482 | 264 | 1.4e-69 |
| AAF08795.1 | MycA | BGC0001103 | Mycosubtilin\_biosynthetic\_gene\_cluster | 32 | 20.9664848012 | 262 | 5.3e-69 |
| CBW45692.1 | putative\_modular\_polyketide\_synthase\_(fragment) | BGC0000952\_c3 | Pristinamycin\_biosynthetic\_gene\_cluster | 33 | 20.6547155105 | 262 | 5.3e-69 |
| CAL69888.1 | RhiA\_protein | BGC0001112 | Rhizoxins\_biosynthetic\_gene\_cluster | 31 | 20.8885424786 | 254 | 1.4e-66 |
| BAF50721.1 | hybrid\_non\_ribosomal\_peptide\_synthetase-polyketide\_synthase | BGC0001116 | Virginiamycin\_biosynthetic\_gene\_cluster | 32 | 20.1870615744 | 251 | 9.3e-66 |
| AMY15068.1 | hexaketide\_synthase\_MF-SQHKS | BGC0001339 | Squalestatin\_S1\_biosynthetic\_gene\_cluster | 28 | 34.7622759158 | 247 | 1.8e-64 |
| XP\_001220460.1 | hypothetical\_protein | BGC0001182 | Chaetoglobosins\_biosynthetic\_gene\_cluster | 29 | 24.2400623539 | 229 | 3.8e-59 |
| CAJ76285.1 | putative\_polyketide\_synthase | BGC0000972 | Colibactin\_biosynthetic\_gene\_cluster | 31 | 21.3561964147 | 224 | 1.6e-57 |
| CAG23961.2 | polyketide\_synthase | BGC0001089 | Bacillaene\_biosynthetic\_gene\_cluster | 29 | 21.395167576 | 223 | 2.1e-57 |
| AGC65516.1 | TtbA | BGC0001050 | Thalassospiramide\_biosynthetic\_gene\_cluster | 30 | 20.3819173811 | 212 | 4.8e-54 |
| CCM44330.1 | putative\_polyketide-type\_polyunsaturated\_fatty\_acid\_synthase | BGC0001056 | Zeamine\_biosynthetic\_gene\_cluster | 27 | 28.9945440374 | 210 | 1.8e-53 |
